# The short cytoplasmic region of phage T4 holin is essential for the transition from impermeable membrane protein complexes to permeable pores

**DOI:** 10.3389/fmicb.2025.1579756

**Published:** 2025-05-30

**Authors:** Jan Michel Frederik Schwarzkopf, Ruth Paola Viveros, Ali Nazmi Burdur, Denise Mehner-Breitfeld, Natalia Tschowri, Thomas Brüser

**Affiliations:** Institute of Microbiology, Leibniz Universität Hannover, Hanover, Germany

**Keywords:** holins, membrane proteins, phages, phage lysis, endolysins, *Escherichia coli*

## Abstract

Virulent as well as temperate phages usually require lysis of bacteria to release their progeny into the surrounding. Double-stranded DNA phages achieve lysis by phage-encoded endolysins that degrade the bacterial cell wall. Endolysins cross the cytoplasmic membrane by the aid of phage-encoded hole-forming membrane proteins, the holins. Canonical holins have been shown to multimerize and form very large holes in membranes, and it is believed that these holes enable a non-specific release of endolysins from cytoplasm. We studied these aspects with *Escherichia coli* and the phage T4 model lysis system, consisting of the holin T and the endolysin E. By following the endolysin function in a microfluidic chamber with mEGFP-fused holin, we found that large multimerizations were not required for endolysin release. Moreover, while we found in further analyses of this construct that the periplasmic globular domain was not required for hole formation and thus likely serves only regulatory functions, the short cytoplasmic domain was essential for hole formation. A truncation as well as single point mutations abolished hole formation without affecting holin interactions. In agreement with this, AlphaFold 3 indicates that rings of holin T dimers can form aqueous holes that require a conformational switch of the N-terminal cytoplasmic amphipathic helix to a trans-membrane orientation inside the rings. Our data indicate that already small holin assemblies enable endolysin-mediated cell lysis, although large multimers may be formed in the absence of endolysins due to the clustering of such assemblies. Further, we provide a convincing structural model of a ring-shaped holin T complex that can form an aqueous pore of sufficient diameter to permit endolysin release.

## 1 Introduction

Most phages release their progeny into the surrounding by lysing the infected bacterial host cell. To achieve this, phages with double-stranded DNA genomes degrade the cell wall by the activity of phage-encoded endolysins (Jacob and Fuerst, 1958). These small proteins reach the bacterial cell wall by holins, which are hole-forming self-interacting membrane-proteins (Young, 1992). In Gram-negative bacteria, the cells are lysed explosively by an additional destruction of the outer membrane, mediated by phage-encoded spanins (Summer et al., 2007). Without spanins, cells growing under conditions without shearing forces, which are most natural conditions, would not lyse. However, in shaking cultures, these spanins are not essential for lysis phenotype due to the shearing forces (Berry et al., 2013).

Diverse classes of holins were described for different phages. While canonical holins form holes that are large enough for the passage of endolysins, pinholins form only small holes that suffice to depolarize membranes, which triggers a release and activation of previously Sec-dependently translocated membrane-anchored endolysins (Young, 2002).

Phage T4 is a model system for the analysis of lysis as mediated by the canonical holin T in conjunction with the endolysin E, a lysozyme that hydrolyzes the β-(1–4)-glycosidic bond between *N*-acetylmuramic acid and *N*-acetylglucosamine in cell wall murein (Kuroki et al., 1993). This phage can mediate an explosive cell lysis after polar or lateral leaks of cytoplasmic content or after formation of spherical cells that can easily burst in the presence of shearing forces, as their cell wall is degraded and only their outer membrane is still intact (Mandal et al., 2021). In the absence of endolysins, the production of the holin can generate holes with diameters up to several hundred nm, and it has been suggested that these large holes are the basis of an unspecific endolysin release (Savva et al., 2014). However, it is currently unclear whether endolysins can already be released earlier by smaller holin assemblies.

No mono- or oligomeric structures of full-length holin T could be revealed so far, but the structure of the periplasmic domain could be partially resolved when its aggregation was prevented by an interaction with the holin-inactivating antiholin RI (Krieger et al., 2020). This antiholin serves to delay lysis in environments with high phage titer (lysis inhibition or LIN, Doermann, 1948; Abedon, 2019), which is postulated to be sensed by binding of the RI/holin complex to periplasmic phage DNA (Krieger et al., 2020). An association of two T/RI dimers was observed in these experiments, but earlier and later studies showed that T/RI dimers represent the physiologically important antiholin-inhibited complex (Moussa et al., 2012; Schwarzkopf et al., 2024). All this indicates that the interaction with the antiholin, which is mediated by the globular periplasmic domain, prevents the oligomerization of the holin to any functional assembly. Previously, mainly non-directed mutagenesis screens identified missense mutations that negatively affect the lysis function of T (Ramanculov and Young, 2001b; Moussa et al., 2014). In addition, a point mutation in the amphipathic helix has been generated by site directed mutagenesis that accelerates the holin function (Ramanculov and Young, 2001b), and cysteine exchanges have been made by directed mutagenesis, which demonstrated a key role of the periplasmic disulfide bond (Moussa et al., 2012).

Although the mechanism of endolysin transport is not entirely understood, it is generally assumed to be similar in the highly diverse and phylogenetically unrelated canonical holins. Fluorescence microscopic analyses of GFP-labeled holin S from the well-characterized λ phage revealed the assembly of large holin aggregates (White et al., 2011). Recently, it has been demonstrated by single cell microscopy that lysis is initiated near these assemblies (Cahill et al., 2024). Based on these data, a model was proposed in which holins harmlessly multimerize to raft-like structures that—upon some stimulus such as a membrane depolarization—can rearrange to a single hole (Cahill et al., 2024). Using holin T of phage T4, we now provide evidence that smaller assemblies likely suffice to permit the release of endolysins, and that the N-terminal amphipathic helix plays a key role in the formation of aqueous pores that mediate endolysin release. Current AlphaFold 3 predictions point to a topological transition as basis for formation, and this transition is triggered by a ring formation when linear holin assemblies are long enough.

## 2 Materials and methods

### 2.1 Strains and growth conditions

*Escherichia coli* strain ER2566 (NEB, Ipswich, United States) was used for protein production in all physiological and biochemical analyses. *E. coli* strain ER2566 is a B/K12-hybrid strain that lacks the DE prophage and any lytic functions (Fomenkov et al., 2017). *E. coli* XL1-Blue Mrf’ Tet (Stratagene), or DH5αλ *pir* + (Invitrogen) were used for cloning. Cells were grown aerobically in LB medium (1% tryptone, 0.5% yeast extract, 0.5% NaCl) at 37°C with appropriate antibiotics (100 μg/ml ampicillin, 25 μg/ml chloramphenicol). P*_*rhaB*_*-dependent gene expression was induced with 0.1% rhamnose and 0.5% glucose was added to overnight cultures for the repression of gene expression. Note that holin production was mediated by the same rhamnose-inducible vector system in all experiments, including physiological as well as biochemical analyses.

### 2.2 Plasmids and genetic methods

Monomeric EGFP (mEGFP) was generated by an A206K exchange (Zacharias et al., 2002), using the primers EGFP-A206K-F and EGFP-A206K-R with the *EGFP* gene from pEGFP-N1 (Clontech, United States) as template, resulting in pEGFP-N1(A206K). The genes encoding mEGFP and T were fused by overlap extension PCR with primers F1-EGFP-F and F1-EGFP-T-R for the mEGFP-coding region, with pEGFP-N1(A206K) as template, and F2-EGFP-T-F and F2-T-R for the holin-coding region, with the holin gene from pBW-*t*-H6 as template (Schwarzkopf et al., 2024). The fusion was cloned into pBW(OT2), which is identical to pBW22 (Wilms et al., 2001), except an additional *Xho*I site in the multiple cloning site, resulting in pBW-*mEGFP-t*, which was used for production of mEGFP-fused holin T (see Supplementary Figure 1 for a plasmid map of this vector system that was used throughout this study for all holin constructs). For production of mEGFP alone with pBW-*mEGFP*, the *mEGFP* gene was amplified from pEGFP-N1(A206K) with the primers F1-EGFP-F and EGFP-*Xho*I-R, and cloned into pBW(OT2). Amino acid exchanges were generated by site directed mutagenesis, using either QuikChange^®^ (Stratagene) or Q5^®^ Site-Directed Mutagenesis (Biolabs), with pBW-*mEGFP-t* as template. Vectors encoding FLAG^®^ -tagged full-length and C-terminally truncated variants of mEGFP-T were constructed by amplifying the corresponding sequence from pBW-*EGFP-t* using F1-EGFP-F as forward and an individual reverse primer. The resulting products were restriction-cloned into pBW(OT2). pBW-*mEGFP-t*(Δ22N), which encodes the holin T with a truncation of 22 N-terminal amino acids, was constructed by overlap extension PCR after amplification of the first fragment with primers F1-EGFP-F and F1-EGFP-T(Δ22N)-R and the second fragment with F2-EGFP-T(Δ22N)-F and F2-T-R, using pBW-*EGFP-t* as template in both PCRs. As in the other constructs, the fusion was cloned with *Nde*I and *Xho*I into the corresponding sites of pBW(OT2). Note that in this study all holin variants were produced by use of the above describe pBW22-based rhamnose-inducible expression systems, which have the ColEI/pMB1 origin (ca. 20 copies/cell, Clewell and Helinski, 1972). All used primers are listed in Table 1. All constructs were confirmed by sequencing. The T4 endolysin was produced using the vector pLysS-*t4l*, a previously reported derivative of pLysS in which the open reding frame (ORF) encoding the T7 endolysin was exchanged by the ORF encoding the T4 endolysin (Schwarzkopf et al., 2024), and the corresponding empty vector pΔLysS was used for control experiments without endolysine (Schwarzkopf et al., 2024). These vectors have p15A origins (ca. 15 copies/cell, Hiszczyńska-Sawicka and Kur, 1997).

**TABLE 1:** Primers used in this study.

Primer names	Sequence (5′ > 3′)
EGFP-A206K-F^1^	CTGAGCACCCAGTCCAAGCTGAGCAAAGACCC
EGFP-A206K-R^1^	GGGTCTTTGCTCAGCTTGGACTGGGTGCTCAG
F1-EGFP-F	ATATACATATGGTGAGCAAAGGCGAGGAG
F1-EGFP-T-R	CTAGGTGCTGCCGGTCTCTTGTACAGCTCGTCCATGC
F2-EGFP-T-F	GACGAGCTGTACAAGAGACCGGCAGCACCTAGAA
F2-T-R	TATATCTCGAGTTATTTAGCCCTTCCT
EGFP-*Xho*I-R	GATATCTCGAGTTACTTGTACAGCTCGTCCATGC
T199-flag-R	TATATCTCGAGTTACTTGTCGTCATCGTCTTTGTAGTCACTTATATGATCATTTCTAT
T148-flag-R	TATATCTCGAGTTACTTGTCGTCATCGTCTTTGTAGTCTCCATTTAAATGAACTGTAT
T93-flag-R	TATATCTCGAGTTACTTGTCGTCATCGTCTTTGTAGTCAGATGATATATGAACTATCTG
T53-flag-R	TATATCTCGAGTTACTTGTCGTCATCGTCTTTGTAGTCACTATCTCCCCTATACCAAAC
T-flag-R	TATATCTCGAGTTACTTGTCGTCATCGTCTTTGTAGTCTTTAGCCCTTCCTAATATTC
F1-EGFP-TΔ22N-R	GGTAGCGTTATCTTTCTTGTACAGCTCGTCCAT
F2-EGFP-TΔ22N-F	GACGAGCTGTACAAGAAAGATAACGCTACCGGGAA
t-F22A-F^2^	AGATCGCTTGGCCAAAGATAACG
t-F22A-R^2^	AGAACACCAAATAGAATATCAG
t-R34A-F^2^	GGAAGGTTCTTGCTTCCGCGGTAG
t-R34A-R^2^	CTACCGCGGAAGCAAGAACCTTCC

^1^Primers for Q5^®^ Site-Directed Mutagenesis.

^2^ Primers for QuikChange^®^ Mutagenesis.

### 2.3 Biochemical methods

Cells from 100 mL cultures were harvested by centrifugation (3,260 × g, 4°C, 10 min) and normalized to 100 ml of an OD600 of 1. Cells were resuspended in 4 ml 50 mM Bis-Tris HCl, 20% (w/v) sucrose, 10 mM MgCl_2_, pH 7.0, and membrane fractions were prepared by two French pressure cell passages (900 psi), followed by a low speed centrifugation (20,000 × g, 10 min, 4°C) to separate cell debris, and a high speed centrifugation (130,000 × g, 30 min, 4°C) to sediment the membranes. Membranes were resuspended in 200 μL 1% *n*-dodecyl-β-D-maltopyranoside (DDM), and native membrane proteins were solubilized for 1 h on ice. SDS-PAGE and Western blotting were carried out according to the standard protocols, with 5 μL of samples analyzed in each lane, and 100 V (Laemmli, 1970; Towbin et al., 1979). Native PAGE was performed in the same way but without DTT or SDS in the buffers and gels. Blots were blocked with 5% skim milk in 137 mM NaCl, 2.7 mM KCl, 4.3 mM Na_2_HPO_4_, 1.5 mM KH_2_PO_4_ (phosphate buffered saline, PBS), rinsed 3 × 5 min in PBS, incubated with primary antibodies (anti GFP, antibodies-online.com, product number ABIN3020571, diluted 1:3,000 in PBS) for 1 h, rinsed 3 × 5 min in PBS, incubated for 1 h with secondary antibody (HRP)-conjugated goat anti-mouse antibodies (Invitrogen, product number 31430, diluted 1:10,000 in PBS), rinsed 3 × 5 min in PBS, and developed using the ECL system (GE Healthcare). In-gel fluorescence of EGFP (excitation with 488 nm laser, 520 nm emission filter) was detected using a Typhoon Trio scanner (Amersham Biosciences).

### 2.4 Fluorescence microscopy

Time-lapse analyzes were performed using a CELLASIC^®^ ONIX B04A-03 Microfluidic Plate (Merck Millipore), in which the cells in a contiuous flow of LB medium were trapped in an area with height of 0.9 or 1.1 μm. For propidium iodide (PI) staining, 1 ml of the cultures were sedimented by centrifugation (7,000 × g, 3 min), resuspended in 500 μl 1 μg/ml PI in PBS (stock solution: 2 mg/ml in water, manufacturer: Promokine), incubated for 5 min at 4°C and washed with PBS. Stained cells were analyzed on agarose slide (1% agarose in PBS). Images were captured with a DMI8 Thunder microscope (Leica), in combination with a Leica-DFC9000GT camera and an HC PL APO 100×/1.40 OIL objective. The filters of a DFT51010 cube were used for the fluorescence of EGFP (475 nm excitation, 519 nm emission, exposure time 300 msec, 50% LED power) and PI (555 nm excitation, 594 nm emission, exposure time 200 msec, 100% LED power). To exclude background fluorescence as detected without production of mEGFP, the tonal range was identically adjusted for all fluorescence micrographs.

### 2.5 Computational methods

The web service AlphaFold Server was used for the prediction of protein structures and for the generation of complex models, using the version AlphaFold 3 (Abramson et al., 2024). Structure images were generated using ChimeraX (Meng et al., 2023).

## 3 Results

### 3.1 Small assemblies of holin T can mediate endolysin transport

**FIGURE 1:**
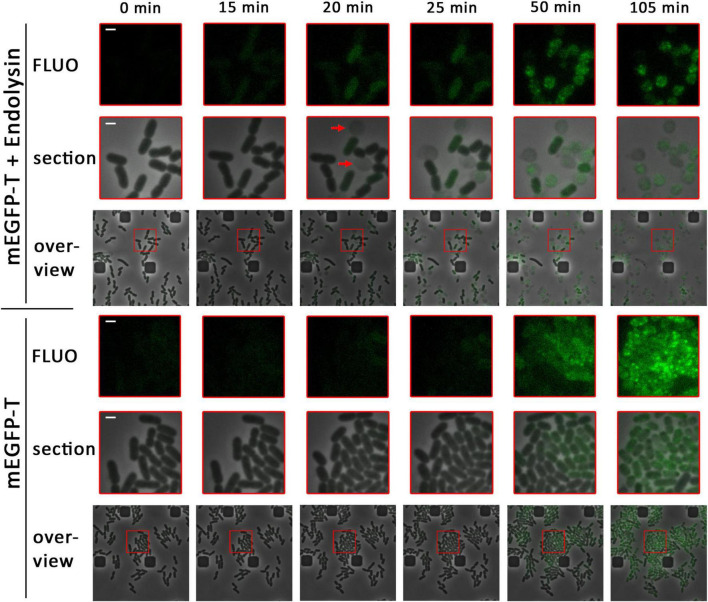
Already small associations of holins enable endolysin release. Detection of mEGFP-fused holin (pBW-*mEGFP-t*), produced by rhamnose induction for the indicated time spans in the presence (pLysS-*t4l*) or absence (empty vector pΔlysS) of the T4 endolysin, using a fluorescence microscope equipped with a microfluidic cell. Overviews show a larger region with an overlay of phase contrast images and corresponding fluorescence images. Enlarged sections (red squares) of these overviews are shown above the overviews, as well as the fluorescence micrographs of these sections (FLUO). The first formation of spherical cells, which is due to functional endolysin release, is indicated by red arrows. The scale bar corresponds to a length of 1 μm.

To examine whether previously observed large holin-aggregates are actually required for hole formation and the endolysin passage, we monitored in a microfluidics system the localization of holins and the endolysin-dependent formation of spherical cells in the *E. coli* strain ER2566 with or without constitutive endolysin production, using a rhamnose-inducible expression system for the production of an mEGFP-fused holin T (Figure 1). The fluorescent mEGFP, a monomeric variant of EGFP, was fused to the N-terminus of the holin, as C-terminal fusions have been reported to markedly reduce the holin function (Ramanculov and Young, 2001a; Ramanculov and Young, 2001b).

In the presence of the T4 endolysin, first spherical cells were observed 15–20 min after induction of the production of the mEGFP-holin fusion, indicating functional endolysin transfer into the periplasm and thus holin function at this early time point (Figure 1A). Cell wall degradation coincided with the emergence of low and diffuse mEGFP fluorescence, which preceded by ca. 30 min the formation of larger mEGFP clusters. After cell wall degradation, mEGFP-holin fusions remained inside the spherical cells and clustered into few larger foci. In conclusion, the data indicated that small assemblies of holin T must have mediated endolysin transport and lysis, as no large associations of holins could be detected at the onset of functional endolysin transport. When fluorescence in these micrographs was not reduced to a level that excludes background fluorescence, it became clear that a subset of the cells had started the production of the holin after 20 min of induction, which was the time point of first functional endolysin transport, and that many small mEGFP-holin signals were evenly distributed in these cells (Supplementary Figure 2).

In the absence of endolysin, no lysis was observed and first very weak fluorescence was detected ca. 25 min after induction (Figure 1B). Fluorescence foci occurred ca. 50 min after induction, indicating formation of large clusters of mEGFP-T at later time points. With time, the fluorescence signal was further compressed into few larger foci inside the cells. Notably, growth and cell division was strongly reduced as soon as holin-mEGFP production was detectable, suggesting that holin assemblies were generated in the absence of endolysin that affected membrane proton gradients and thus cellular energetization.

As a control, a strain was equally analyzed that produced the T4 endolysin and mEGFP alone without fused holin (Supplementary Figure 3). As expected, the endolysin was not exported and the cells remained rod-shaped. Evenly distributed mEGFP fluorescence emerged ca. 15–20 min after induction. Fluorescence intensity continuously increased later on, reflecting continued protein synthesis, and cell proliferation was unaffected, indicating that the endolysin and mEGFP alone did not affect the cellular energetization.

### 3.2 The cytoplasmic domain of T is not required for multimerization but essential for membrane permeabilization

**FIGURE 2:**
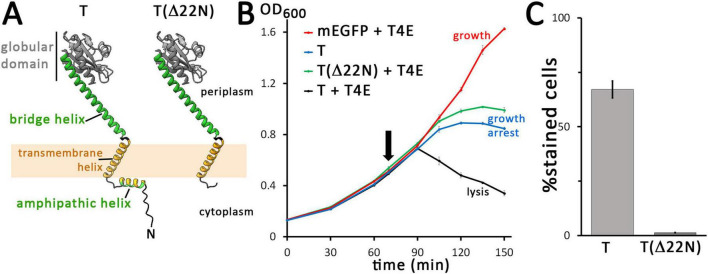
The N-terminal domain of the holin is required for hole formation. **(A)** Structures of the wild type (wt) and N-terminally truncated (Δ22N) variant of the holin T, as predicted by AlphaFold 3. The amphipathic helix is colored in green (polar residues) and orange (hydrophobic residues), and the transmembrane helix is colored in orange. The globular C-terminal domain is colored in gray and the bridging helix that connects this globular domain to the membrane is colored in green. For clarity, tags are not shown. **(B)** Growth curves demonstrating the lysis defect due to the truncation of the N-terminal 22 residues of the holin. The time point of holin gene induction is indicated (arrow). Black, wt holin together with T4 endolysin (T + T4E), which is a positive control that shows lysis in the presence of functional holins and endolysins; green, truncated holin together with T4 endolysin (T(Δ22N) + T4E); blue, control with the holin alone (with the endolysin production vector pLysS-*t4l* substituted by the empty vector pΔlysS), showing that holins can cause a growth arrest but not lysis in the absence of endolysins (T); red, control with mEGFP and T4 endolysin, showing that any lysis required the holin (mEGFP + T4E). Data points and standard deviations are from three technical replicates. **(C)** Diagram showing the portion of cells stainable by propidium iodide after 1 h of production mEGFP-fused wild type T or the truncated variant T(Δ22N). Standard deviations were derived from counts of three survey micrographs. Note that in the absence of the N-terminal 22 residues, membrane permeabilization by holins is blocked. All holin variants analyzed in these experiments were fused to mEGFP, as these analyses characterized the system analyzed in Figure 1, and all holin expression was from the rhamnose-inducible pBW systems.

The domain structure of the phage T4 holin T is unique among the holins (Ramanculov and Young, 2001b). It is predicted by AlphaFold to consist of a short amphipathic helix in the N-terminal cytoplasmic domain, a single transmembrane helix, a C-terminal globular domain, and a “bridge-helix” that connects the transmembrane helix with—and reaches into—the C-terminal globular domain (Figure 2A; Abeysekera et al., 2022). We used recombinantly generated protein truncations to approach the potential roles of the cytoplasmic (N-terminal) and periplasmic (C-terminal) domains of the holin. As our analyses addressed the function of the mEGFP-fused holin that was capable of endolysin transport without showing any formation of large aggregates or rafts (Figure 1), we studied all truncations or other mutations of the holin with the same N-terminal mEGFP-tag, and we used the mEGFP-fused full-length holin as positive control for holin function and mEGFP alone as negative “no-holin control.” In case of the analyses of the N-terminal domain, we deleted the first 22 amino acids of holin T. Notably, this Δ22N-variant did not mediate lysis in the presence of the T4 endolysin, and it caused a growth arrest phenotype (Figure 2B). These results indicated that holes of sufficient size for endolysin release are not generated by this construct, while there is still a membrane damage by a non-functional aggregation of holins, which likely causes energy limitation due to a reduced proton gradient. In control experiments, we confirmed that the growth arrest was indeed independent of the endolysin (Supplementary Figure 5A). As the fluorescent dye propidium iodide stains cells with permeabilized membranes (Boulos et al., 1999), we monitored membrane permeabilization by this stain. Notably, when produced for 1 h, wild type mEGFP-T fusions permeabilized 67% of the cells (249 counts), whereas only 1.3% of the cells (235 counts) were permeabilized by mEGFP-TΔ22N, indicating that abolishment of propidium iodide permeability correlates with abolished ability to release endolysin (Figure 2C). Notably, foci of mEGFP fluorescence of the mEGFP-T constructs were also formed by the truncated variant, suggesting that these proteins still interacted (Supplementary Figure 4).

**FIGURE 3:**
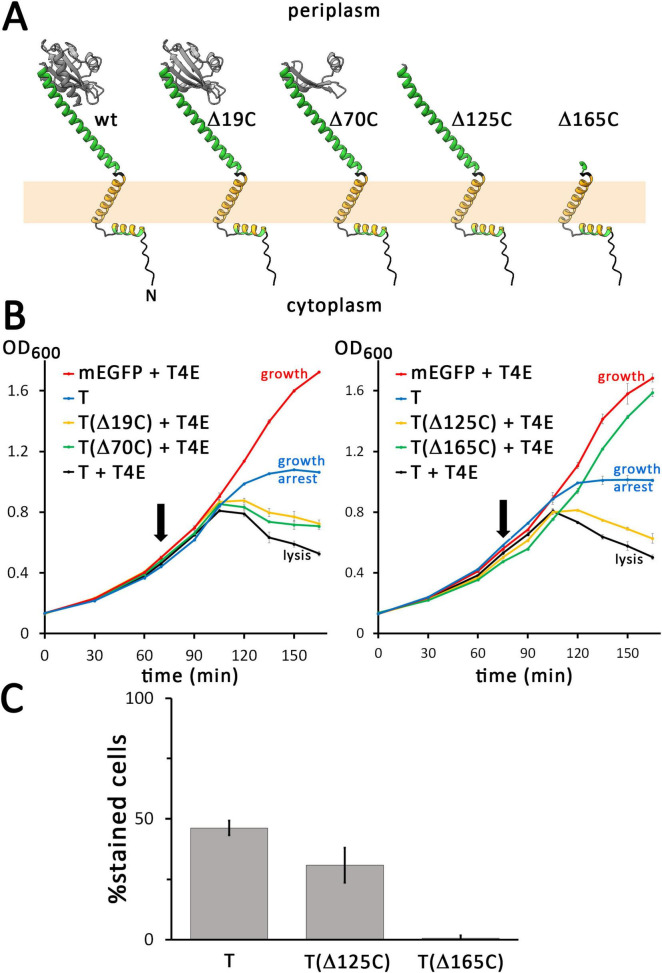
Only the bridge helix of the C-terminal domain of the holin is required for hole formation. (A) Structures of the wild type (wt) and indicated C-terminally truncated variants of the holin T, as predicted by AlphaFold 3. **(B)** Growth curves demonstrating that lysis is still mediated by truncations that contain the bridge helix, but not by the construct that lacks this helix. The tested truncated holin variants are indicated in the respective blots. Where indicated, the T4 endolysin was either constitutively produced using the vector pLysS-*t4l*, or the empty vector pΔlysS was used. **(C)** The membrane permeability mediated by mEGFP-fused FLAG-tagged holins was analyzed with wild type holin T, the smallest functional truncation T(Δ125C), and the non-functional truncation T(Δ165C). Note that all analyzed holin constructs for this analysis were N-terminally fused to mEGFP. In addition, these constructs were C-terminally FLAG-tagged to confer identical C-termini to all constructs, which is important for periplasmic stability (see text for details). As throughout this study, all holin constructs were produced from rhamnose-inducible pBW systems. Note that removing the bridge-helix abolishes hole formation.

To examine the function of the larger periplasmic C-terminal domain of T, we generated several C-terminal truncations, based on the AlphaFold 3 structure prediction. In the periplasm, a long helix connects the transmembrane helix with a globular domain, whose structure has been solved, which agrees with the AlphaFold 3 structure (Krieger et al., 2020). We analyzed the effect of truncations by 19, 70, 125, and 165 residues (Figure 3A). While the 19-residues truncation (Δ19C) lacks a C-terminal helix at the surface of the globular periplasmic domain, the 70-residues-truncation (Δ70C) lacks large parts of the globular domain, the 125-residues truncation (Δ125C) removes the complete globular domain, and the 165-residues-truncation (Δ165C) removes also the long helix between the membrane anchor and the globular domain (Figure 3A). We placed—specifically for the analysis of C-terminal truncations only—the highly charged FLAG tag (DYKDDDDK) at the C-terminus of the truncations and the full-length mEGFP-T control, as the periplasmic protease DegP is known to prefer proteins with hydrophobic C-termini for degradation (Kim et al., 2018). FLAG-tagged full-length mEGFP-T was used as a control. The C-temini of all constructs used for this specific analysis are shown in Supplementary Figure 6. The Δ19C and Δ79C truncations caused only a slight delay in lysis (Figure 3B). Holin function was remarkably tolerant to truncations, as also the Δ125C truncation mediated lysis. However, the Δ165C truncation that lacks also the long helix connecting the membrane anchor with the globular domain was inactive, and the cells showed undisturbed growth similar to the control without the holin. As expected, in the absence of the endolysin, growth was arrested by all constructs with the exception of the Δ165C truncation (Supplementary Figures 5B,C).

The membrane permeabilization was slightly impaired by the Δ125C truncation construct, which showed 30.9% PI-stained cells (368 counts) compared to 46.3% (164 counts) in case of the wild type (Figure 3C). Note that the FLAG tag reduced to some extent the membrane permeabilization function in comparison to the non-tagged construct (see Figure 2C), but the data still clearly showed functionality with respect to hole formation. However, in case of the Δ165C truncation construct, only 0.6% of the cells (296 counts) were stained, demonstrating that the bridge helix that connects the membrane anchor with the globular domain is essential for holin function. All C-terminally truncated variants caused foci of mEGFP fluorescence but the foci of Δ165C construct were larger and localized at the poles, indicating a strong aggregation via the remaining transmembrane helix (Supplementary Figure 7).

**FIGURE 4:**
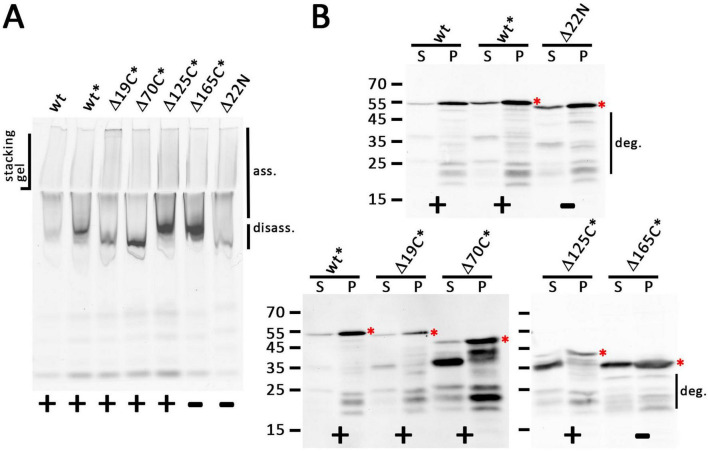
All truncated holin variants can form large associations. **(A)** In-gel fluorescence of mEGFP fusions to indicated holin T variants as produced from the rhamnose-inducible pBW systems after native PAGE, showing a smear of large assemblies (ass.) that can be detected up to the slots of the stacking gel. Note that especially C-terminal truncations cause stronger bands at the lower edge of this broad signal, indicating partial disassembly (disass.) due to destabilization and possibly the influence of incompletely degraded portions of the proteins. **(B)** SDS-PAGE/Western blot detection of wild type and all truncated variants in solubilized membranes (S) and solubilization-resistant holins that sedimented in pellets after solubilization (P). Detection with anti GFP antibodies. The red asterisks indicate the full-length proteins. The region with degradation bands is indicated on the right of the respective blots. In **(A,B)**, the black asterisks mark constructs with C-terminal FLAG tags. Active (+) and inactive (-) constructs are indicated at the bottom of each panel.

When we analyzed the wild type and the truncated variants by native PAGE after detergent-solubilization using dodecyl maltoside (DDM), we found that all solubilized constructs could form large complexes that were detected at the top of the separating gel and the stacking gel, suggesting that neither the truncation of the cytoplasmic domain nor the removal of the periplasmic domain abolished the association of the holin and possibly formations of higher order oligomers, which agrees with the holin activity that was found for Δ19C, Δ70C, and Δ125C variants (Figure 4A). A likely disassembly band occurred with truncated variants, which became more prominent with larger C-terminal truncations, suggesting that truncations disturbed self-interactions of the holins, even when they did not abolish the activity, supporting the view that the C-terminal globular domain contributes to the stability of holin oligomers, albeit this domain is not essential for function.

A further analysis of the DDM-solubilized proteins and also of the portion of proteins that could not be solubilized was done by SDS-PAGE/Western blotting (Figure 4B). This showed that bands expected for intact proteins were detected in all cases. However, there was also some degradation, especially in case of the solubilized proteins, which was prominent with the Δ70C and Δ125C variants, although the lysis assay had shown that sufficient active holin was present. A prominent degradation band was detected at 36 kDa, the size of the inactive Δ165C variant, and therefore likely this band is due to a portion of the truncated protein in which the complete periplasmic domain was degraded. It should be noted that solubilization might have triggered degradation, as there were more prominent degradation bands in solubilized protein than in the pellet fraction after solubilization. A Western blot that detected the C-terminus of the FLAG-tagged mEGFP-holin fusion blot showed that there was no detectable N-terminal degradation (Supplementary Figure 8). Further, the single full-length protein band in this blot also demonstrated that there was no internal start codon generated by the fusion. Note that the N-terminal methionine of the holin was removed by the N-terminal mEGFP fusion, which was used throughout this study (see Supplementary Figure 9 for the sequences of all constructs).

Together, these data clearly demonstrate that inside the periplasm only the “bridge-helix” that connects the globular periplasmic domain to the transmembrane helix is required for endolysin transport and lysis. This further supports the view that the globular domain is required for regulatory purposes only, i.e., for lysis inhibition by antiholins, a function that has been clearly demonstrated (Schwarzkopf et al., 2024). On the cytoplasmic side of the membrane, the N-terminal amphipathic helix is essential for endolysin transport. This amphipathic helix could in principle switch its orientation from a membrane surface position to a transmembrane orientation, and we focused on this aspect as such a switch was likely to represent a key event for the formation of functional holin pores.

### 3.3 Conserved positions F22 and R34 on the cytoplasmic face of the membrane are involved in the essential function of the N-terminus

**FIGURE 5:**
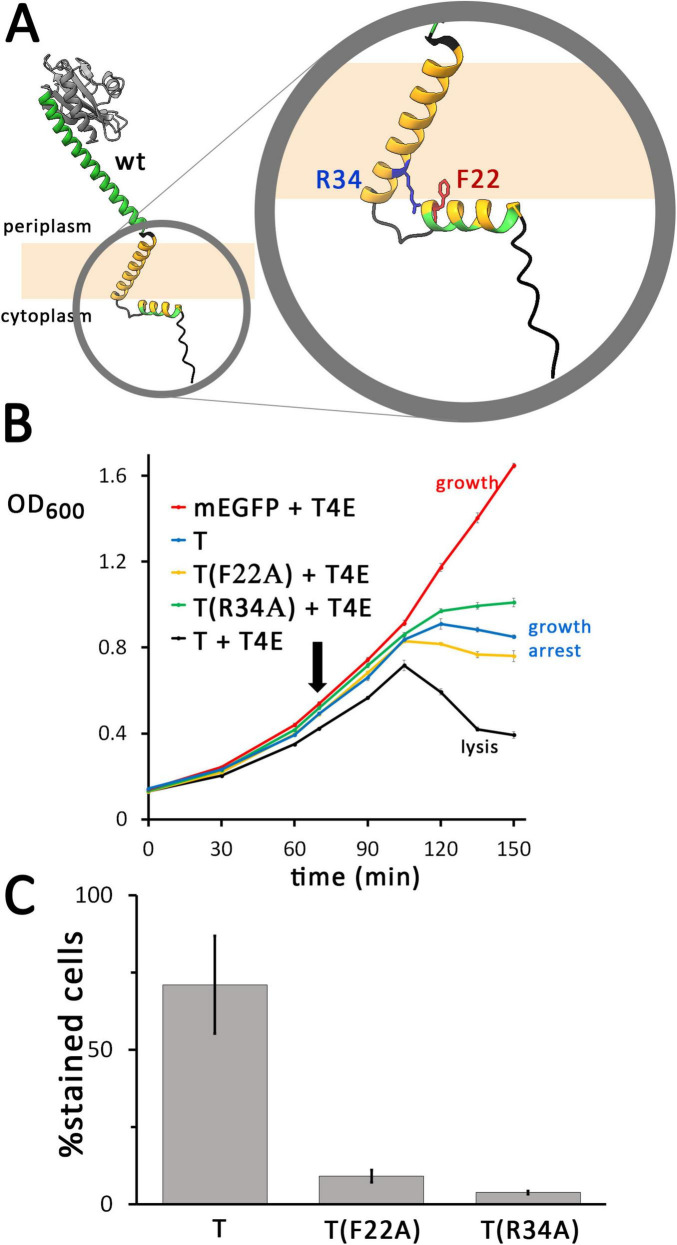
Single mutations in the N-terminal region of the holin can already cause severe hole-formation deficiency. (A) Structures of the wild type (wt) holin T, and enlarged N-terminal region with the mutated positions indicated. **(B)** Growth curves demonstrating that lysis is abolished by the indicated F22A or R34A point mutations of mEGFP-fused holin T. See Figure 2 for further details. As throughout this study, the described rhamnose-inducible holin and constitutive endolysin expression systems were used. **(C)** The F22A and R34A mutations both result in a strong reduction of hole formation ability, as monitored by propidium iodide stain analyses (see Figure 2 for details).

In alignments of closely related holin T homologs, the positions F22 and R34, which are located at the end of the amphipathic helix and the beginning of the transmembrane helix, respectively, are highly conserved (Figure 5A and Supplementary Figure 10). In order to examine the potential importance of these positions, we exchanged these residues by alanines and analyzed the localization and functionality of these constructs (Figure 5B). In a positive control, the wild type holin T caused lysis when the T4 endolysin was present. In contrast, the F22A mutation caused only a growth arrest, similar to a control with wild type holin in the absence of the endolysin. This indicated that the conserved aromatic residue of F22 at the end of the cytoplasmic helix is essential for function. The R34A exchange caused similar effects, suggesting that also the R34 position is important for holin function.

To examine a potential membrane permeabilization by the F22A and R34A holin variants, it was analyzed whether cells producing these variants could be stained by propidium iodide (PI) (Figure 5C). As a control, the wild type holin sequence was included in the analysis. After 1 hour of induction, a large portion of cells producing mEGFP-T were stained (71%, 401 counts) in the wild type control, whereas only 9% (695 counts) of the F22A- and 4% (1,478 counts) of the R34A-variant producing cells were stained. These data show that the region between the cytoplasmic amphipathic helix and the membrane domain of holin T is important for the permeabilization of the membrane. Holin clusters were still formed in all cases, as fluorescent foci of holin clusters were detected in all cultures (Supplementary Figure 11). The signal intensity was increased relative to the wild type in case of the production of F22A variant, and the signal was even stronger when the R34A-variant was produced. The enhanced protein production further supports the view that the F22A or R34A mutations suppress harmful membrane defects that are generated by wild type holins, which likely improves cellular energetization.

**FIGURE 6:**
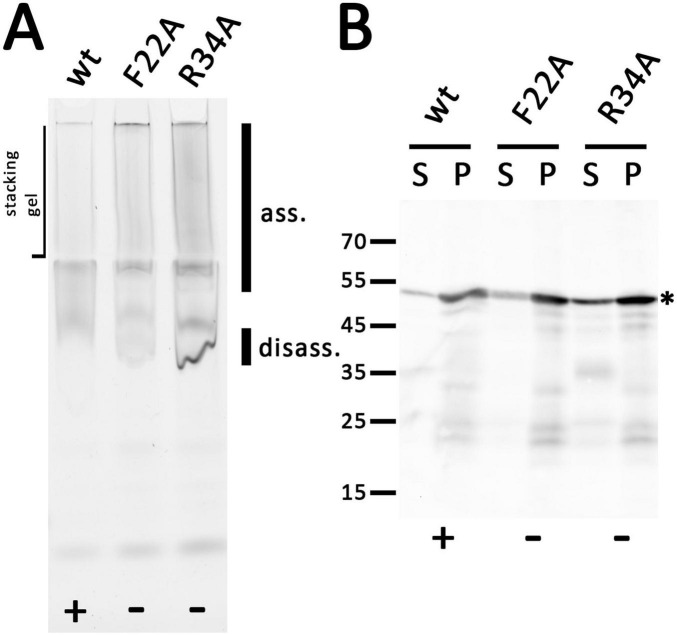
Holin T with the point mutations F22A or R34A still form large associations but influence the assembly similar to truncations. **(A)** In-gel fluorescence of indicated mEGFP fused holin variants after native PAGE. The same rhamnose-inducible pBW expression system was used as throughout this study. The smear of large associations (ass.) is hardly influenced by the point mutations, but there is a significant increase of a disassembly product (disass.). **(B)** SDS-PAGE/Western blot detection of wild type and the two mutated variants in solubilized membranes (S) and solubilization-resistant holins that sedimented in pellets after solubilization (P). Detection with anti GFP antibodies. The asterisk marks the full-length proteins. Active (+) and inactive (-) constructs are indicated at the bottom of each panel.

The ability of the F22A and R34A variants to form homooligomeric or multimeric clusters was also assessed by native PAGE (Figure 6A). Membrane protein complexes of strains producing the wildtype or mutated variants were detergent-solubilized with DDM and analyzed. Bands of large complexes of the wild type holin and the two variants were detected by in-gel fluorescence at the upper end of the separating gel and in the stacking gel, indicating that both lysis-inactive variants formed multimers. A clear band at the lower edge of this diffuse signal was present in case of the F22A and R34A variants, which was much more intense in case of the R34 variant. This band may be attributed to some disassembly of solubilized oligomers to monomers that is triggered by the mutations.

SDS-PAGE/Western blot analysis showed that all holin variants were correctly folded and detected at 50 kDa (Figure 6B). There was only little degradation, which was not enhanced by the mutations. As expected when considering the stronger fluorescence (see Supplementary Figure 11), the signal of the R34A variant was significantly increased.

### 3.4 AlphaFold 3 predicts a transition from linear to circular assemblies that depends on a topological transition of the amphipathic helix

**FIGURE 7:**
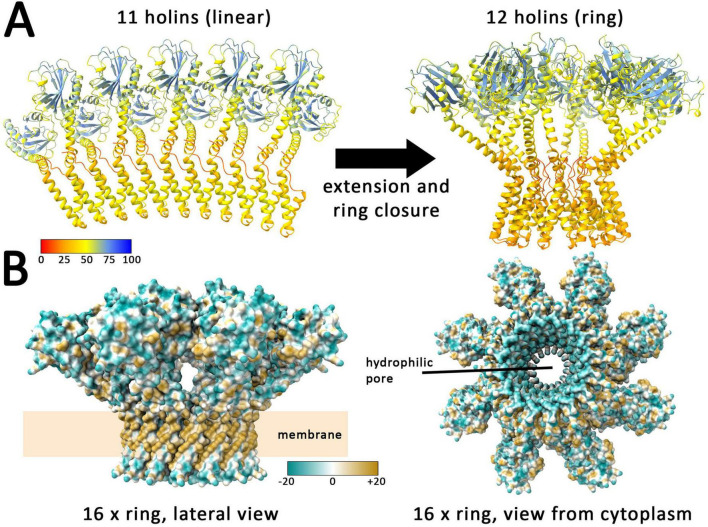
AlphaFold 3 predicts linear assemblies with low numbers of holins and closed rings with 12 or more holins. **(A)** Structures formed by 11 and 12 holins, as predicted by AlphaFold 3. Colors indicate the confidence for the prediction (pLDDT values, see scale), which is usually high for globular folded domains and < 50 for regions that are expected to be flexible, as is the case in the region without tertiary structure, where the holin is anchored to the membrane. **(B)** Lateral and cytoplasmic views on the predicted ring of 16 holin subunits. Surface colored according to hydrophobicity (see scale) with blue indicating hydrophilic and ochre hydrophobic surfaces. Note that the predicted rings expose the expected hydrophobic surface to the surrounding membrane lipids. The inner surface of the ring is hydrophilic, generating an aqueous pore.

In order to elucidate the mechanism of holin-mediated endolysin transport by phage T4, structural insights are of great importance. However, because of its toxic and aggregation-prone characteristics, purifications give low yields and structural characterizations of homooligomeric complexes could not be achieved so far. Only the globular part of the C-terminal domain could be purified and characterized, and this only when a complex with the antiholin prevented aggregation (Moussa et al., 2012; Krieger et al., 2020). However, recent progress in machine learning enabled structure predictions as an alternative approach for analyzing such challenging proteins, especially the highly accurate tool AlphaFold (Jumper et al., 2021). Through the use of AlphaFold 2, convincing models of the holin T monomer and a dimeric interaction with the antiholin RI were previously predicted (Mirdita et al., 2022; Schwarzkopf et al., 2024). The novel AlphaFold 3 now permits the prediction of larger homooligomeric structures with an improved accuracy (Abramson et al., 2024). With up to 11 protomers, AlphaFold 3 predicts linear associations of T4 holins (Figure 7A). Interestingly, modeling of larger complexes composed of 12 or more protomers resulted in rings. Strikingly, in these rings, the N-terminal cytoplasmic amphipathic helix adopts a transmembrane orientation, forming a hydrophilic inner ring surface that would result in an aqueous membrane channel (Figure 7B). It can be expected that without such an orientation, the amphipathic helices would align at the membrane surface and it is likely a coordinated amphipathic helix reorientation in all associated holin protomers that results in hole formation.

Together, AlphaFold 3 models strongly support the interdependence of ring formation and a switch of the amphipathic helix to an transmembrane orientation, which thereby generates an aqueous pore that can suffice to permit the passage of the small endolysins, possibly already with a ring consisting of less than 20 holin protomers. A closer look at the rings reveals that already 16 protomers can generate a hole large enough for the passage of T4 endolysin (Supplementary Figure 12). This fully agrees with our finding that the amphipathic helix is of key importance for hole formation by holins.

## 4 Discussion

**FIGURE 8:**
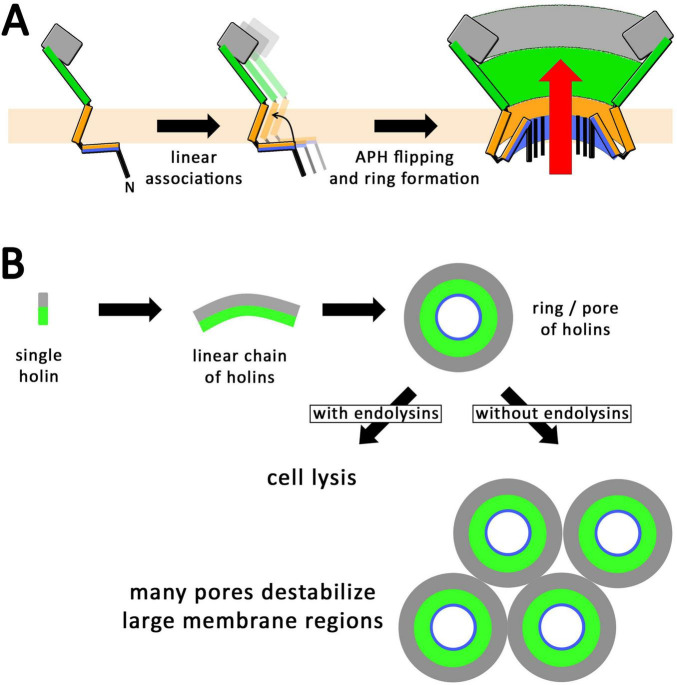
Proposed mechanism for the formation of holin rings. **(A)** Schematic lateral views illustrating the formation of linear holin associations. When the N-terminal amphipathic helix switches in a transmembrane orientation, these linear assemblies must form closed rings with a polar inner pore surface, which permits the passage of endolysins (red arrow). A switch of the amphipathic helix can only occur hand in hand with a ring formation, as otherwise the polar surface of the amphipathic helix would face membrane lipids. **(B)** Scheme for the formation of holin rings, seen from the periplasmic side, which normally—i.e., in the presence of endolysins—leads to lysis with just a few pores (as supported by Figure 1). In the absence of endolysins, many rings could be formed, which could potentially destabilize large membrane regions (as supported by Savva et al., 2014).

Models for the formation of holes by holins have been developed in the past that already took into account that a hydrophilic surface has to be generated by the holins at inner pore surfaces, while hydrophobic helices point toward the membrane lipids (Pang et al., 2009; Cahill et al., 2024). As canonical holins possess entirely hydrophobic transmembrane helices, it was not clear which type of topological change occurs in holin assemblies that can result in such a hydrophilic inner pore surface. In case of the virulent phage T4, one of the most studied model phages, it has already been recognized that the N-terminus of the holin has an amphipathic helix of potentially important function (Moussa et al., 2014). Also some harsh point mutations have been generated in the N-terminal domain by random mutagenesis, which included inactivating positions in the amphipathic helix (D19N, D19G, D19V, L21F, F22S), of which only the F22S variant has been biochemically shown to be stably produced (Ramanculov and Young, 2001b; Moussa et al., 2014). The specific role of the amphipathic helix was not clear, although it was already suggested to be important for hole formation (Moussa et al., 2014). Our inactivating F22A mutation is even a milder exchange than the reported F22S mutation, and our finding that this mutation blocks membrane permeabilization as monitored by propidium iodide staining supports the essential role of the amphipathic helix in hole formation. Also an inactivating R34W mutation had been described, and it was proposed that the positioning of the transmembrane helix by R34 could be important for holin function (Moussa et al., 2014), which agrees with our data, as our inactivating R34A mutation abolished hole formation. We therefore now show that the N-terminal amphipathic helix is indeed essential for the hole formation (Figures 2, 3), and this amphipathic helix is perfectly positioned to interact with the first transmembrane helix. Such a possible interaction suggests a novel mechanism for holin pore formation (Figure 8A): Due to the hydrophobic mismatch that would be generated by a transmembrane orientation of a lipid-exposed amphipathic helix, and the consequent energetic barrier, linear assemblies of holins would not be able to switch the amphipathic helix in a transmembrane orientation. Such a topological switch would only be possible if coupled to a ring closure and lipid displacement, which consequently results in an aqueous hole. In case a ring can already be formed without a transmembrane orientation of the amphipathic helix, it will be hydrophobic inside and thus impermeable for endolysin and propidium iodide until the amphipathic helix adopts a transmembrane orientation. It is the change of amphipathic helix orientation that generates the hydrophilic pore. Our observed endolysin-dependent cell wall degradation at very low holin concentrations (Figure 1) suggests that few such rings likely suffice to cause cell lysis, as endolysins are highly active enzymes that can rapidly degrade cell walls (Figure 8B). The previous observation of micron-scale holes can be caused by holins in the absence of endolysins (Savva et al., 2014), possibly due to lateral associations of multiple holin rings that can destabilize large regions in the cytoplasmic membrane that eventually collapse, being solubilized (Figure 8B).

If this is true, then one would expect that beside the N-terminal cytoplasmic domain and the transmembrane helix only few additional regions would be needed to form such holes, and especially the large periplasmic globular domain would not be required. Indeed, it was already very likely that the globular part of the C-terminal periplasmic domain has at least mainly regulatory functions, as the crystal structure and later biochemical studies showed that the regulatory antiholin interaction is mediated by this globular domain (Krieger et al., 2020; Schwarzkopf et al., 2024). Moreover, a 69-residues C-terminal truncation has been already reported by the Young group to still permit lysis and hence hole formation (Wang et al., 2003). The truncation analyses that we report now are in full agreement with this, as they show that even the complete globular domain can be removed without loss of hole formation activity (Figure 2). Further, our analyses demonstrate that the long helix that connects the transmembrane helix with the C-terminal globular domain is essential for hole formation, which points to a specific contribution of that helix that remains to be understood. Notably, none of the truncations or mutations resulted in a block of self-association to larger assemblies (Figures 4, 6), and large assemblies of inactive holin with single point mutations in the amphipathic helix (position F22) or the arginine residue (R34) that holds the transmembrane helix in place further strengthen the view that it is not the mere multimerization that automatically results in membrane disassembly. It rather appears that a topological switch is required for function that is suppressed by mutations such as F22A or R34A, which supports the view that a topological switch of the amphipathic helix occurs hand in hand with circular hole formation.

In full agreement with all biochemical data, AlphaFold 3 predicts a model for holin oligomers in which the amphipathic helix interacts with the first transmembrane helix. So far, AlphaFold 3 cannot include the effects of a membrane environment, which is why this interaction is predicted also for linear holin assemblies. However, in the presence of the membrane, the amphipathic helix would lay on the membrane surface, which would block the interaction with the transmembrane helix. Notably, linear assemblies are only predicted with up to 11 holin protomers, and rings are formed thereafter, which can differ in size and organization depending on the number of involved protomers, but it is intriguing that a linear association larger than a certain threshold length can spontaneously close to a ring.

Such a ring closure or the topological switch of the amphipathic helix that results in the aqueous pore may be facilitated by a membrane depolarization, which would explain the trigger of hole formation and lysis by PMF-reducing agents (Doermann, 1952). In the natural system, such a membrane depolarization may be already achieved by holin assemblies before they form the holes. Experimental evidence for this comes from our inactive holin variants that do not form holes of sufficient size to permit the passage of the fluorescent dye propidium iodide, but that are able to cause bacterial growth arrest (Figures 2, 5).

Another important aspect is the fact that larger assemblies of holins can in principle result in micron-scale membrane defects (Savva et al., 2014). Such large associations can be seen when holin production continues in the absence of endolysins, and it can be imagined that multiple holin rings can laterally associate in large areas of the membrane, something that has already been proposed for pinholins (Pang et al., 2013), which can in principle result in a local membrane solubilization that is detectable by electron microscopy. We would like to emphasize that these observations are therefore in full agreement with our findings.

It did not escape our attention that the key role of the cytoplasmic amphipathic helix switch allows for a lysis inhibition by antiholins that bind to this helix, and such an antiholin, RIII, has indeed been identified (Chen and Young, 2016), further supporting the view that the inhibition of a topological switch can contribute to lysis inhibition. In future, antiholin mechanisms as well as the holin assemblies prior to pore formation might be studied in our reconstituted system by the membrane permeability-based hole formation assays.

Together, this study clarifies the specific essential role of the short cytoplasmic N-terminal domain and the first periplasmic helix for hole formation, it discriminates depolarization and hole formation by holin assemblies, and it proposes a first molecular mechanistic model for hole formation by T4 holins, which may well apply also for other phage systems. It has to be kept in mind that we analyzed the holin function in a reconstituted lysis system with constitutive endolysin and inducible holin production. In our system, regulation certainly had the limitation to lack the complexity of whole phage infections, which include more regulatory components, but it had the advantage to permit clear attributions of effects to the analyzed holin constructs only. Based on our findings, future studies will hopefully experimentally clarify the structure of the rings and the protomer interactions therein, and their potential specificity for small proteins such as endolysins at the beginning of the lysis process.

## Data Availability

The original contributions presented in this study are included in this article/Supplementary material, further inquiries can be directed to the corresponding author.
