# Supplementary material for: The short cytoplasmic region of phage T4 holin is essential for the transition from impermeable membrane protein complexes to permeable pores
Source: Front Microbiol. 2025 May 30;16:1579756. doi: 10.3389/fmicb.2025.1579756 (PMC12162936; doi:10.3389/fmicb.2025.1579756)
Supplement: Supplementary file 1 [file Data_Sheet_1.pdf]

The short cytoplasmic region of phage T4 holin is essential for the transition from impermeable membrane protein complexes to permeable pores

Jan Michel Frederik Schwarzkopf, Ruth Paola Viveros, Ali Nazmi Burdur, Denise Mehner-Breitfeld, Natalia Tschowri and Thomas Brüser

Supplementary Figures

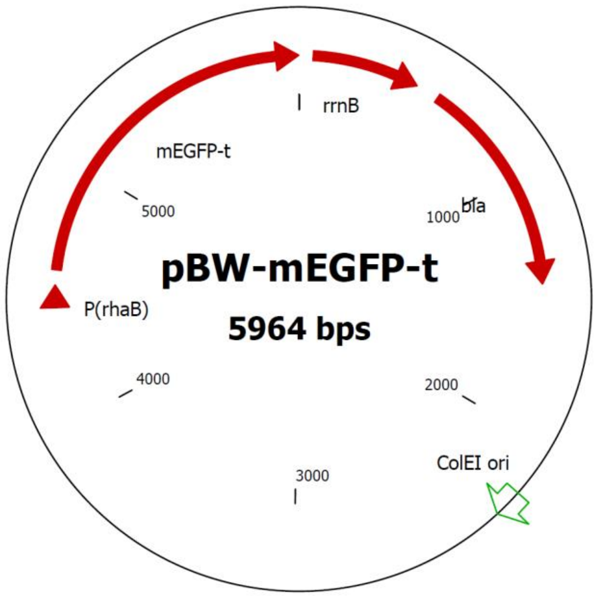

Supplementary Figure 1. Plasmid map of pBW-*mEGFP-t*. Regions of the P_{rhaB} promoter, the coding region for the *mEGFP-t* fusion, the *rrnB* transcriptional terminator, the ampicillin resistance gene (*bla*), and the ColEI origin of replication are indicated.

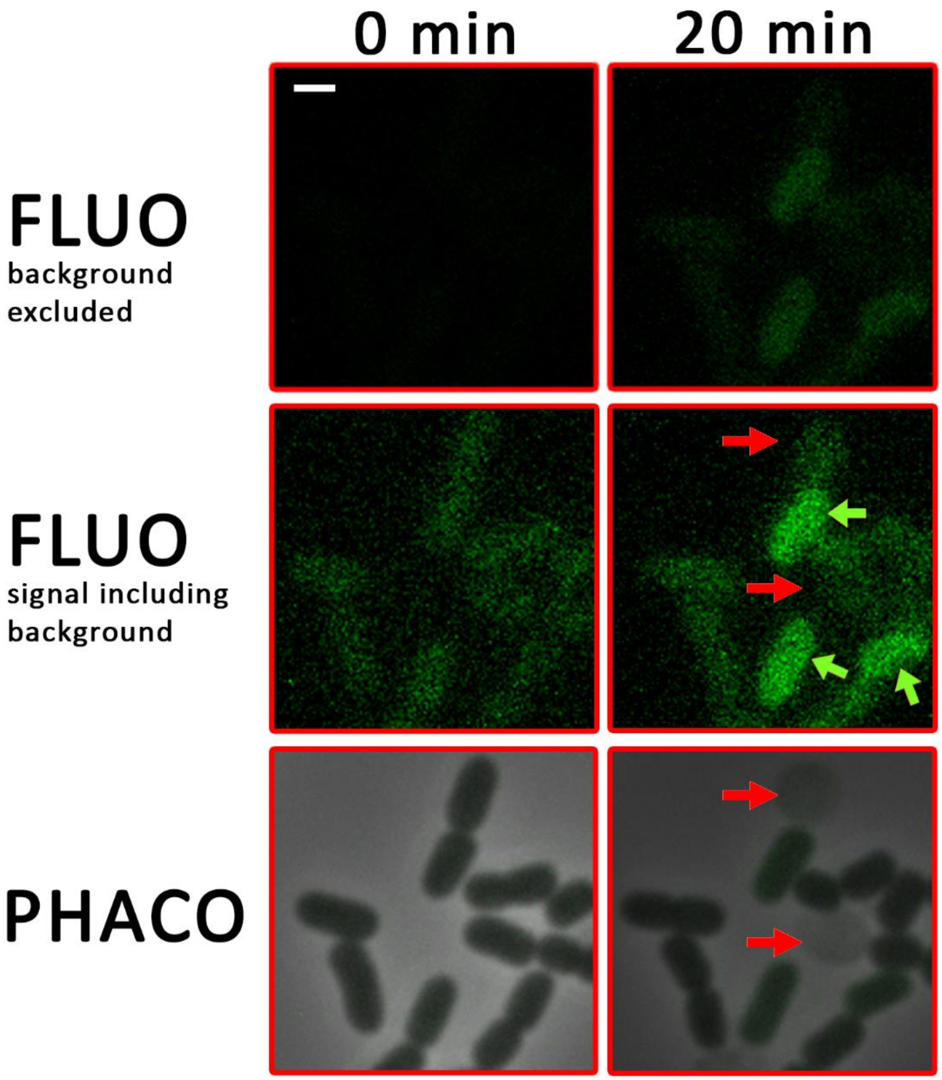

Supplementary Figure 2. At the time point of first mEGFP-holin dependent endolysin transport (20 min, see red arrows that indicate endolysin activity), first mEGFP fluorescence is detected in a subpopulation of cells, and mEGFP fluorescence is evenly distributed. Note that these micrographs are taken from Fig. 1, and only those with residual background fluorescence (middle) are added to show that not all cells had already induced the production of the mEGFP-holin fusion at that time point (green arrows). These micrographs facilitate also the detection of the very low fluorescence after 20 min. Scale bar: 1 µm.

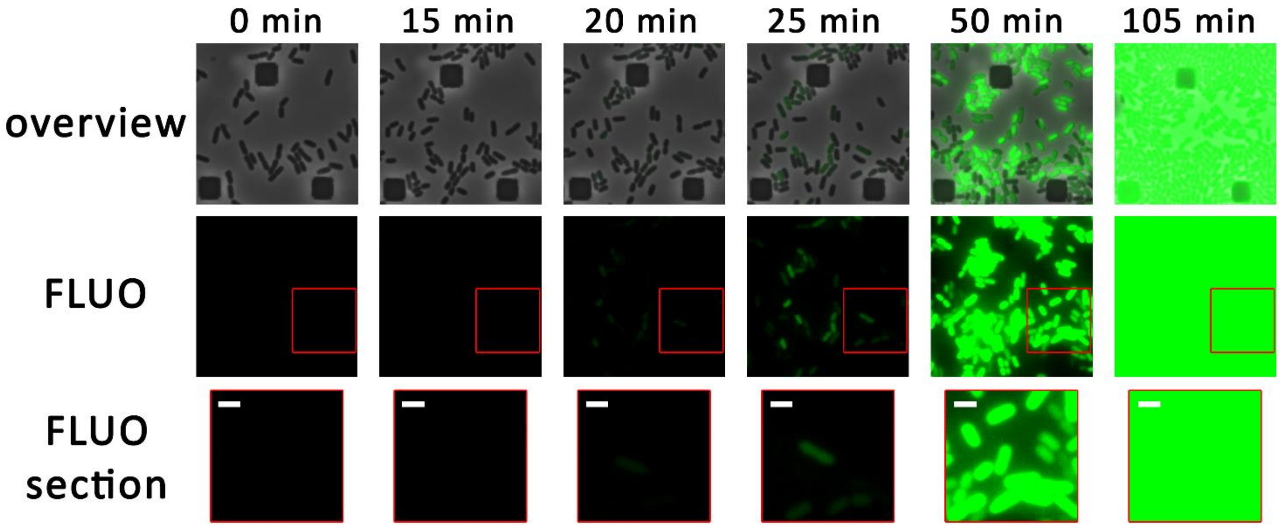

Supplementary Figure 3. Production of unfused mEGFP with the same expression system as used for the holin fusions shown in Figure 1 results in accumulation of very high abundances without formation of foci. Expression was induced by rhamnose as in the experiments shown in Fig. 1. The strain contained T4 endolysin, constitutively produced by pLysS-*t4l*. Overviews show overlay of phase contrast and epifluorescence micrographs, as in Fig. 1. “FLUO” panels show the epifluorescence channel only. The indicated sections (red squares) of these micrographs are shown in the “FLUO section” panels. The scale bare indicates the length of 1 µm. Note that the fluorescence in the “FLUO” and “FLUO sections” is equally and linearly adjusted as in Fig. 1, which is why the fluorescence intensity at time point 105 min is extremely high, not permitting the recognition of single cells anymore.

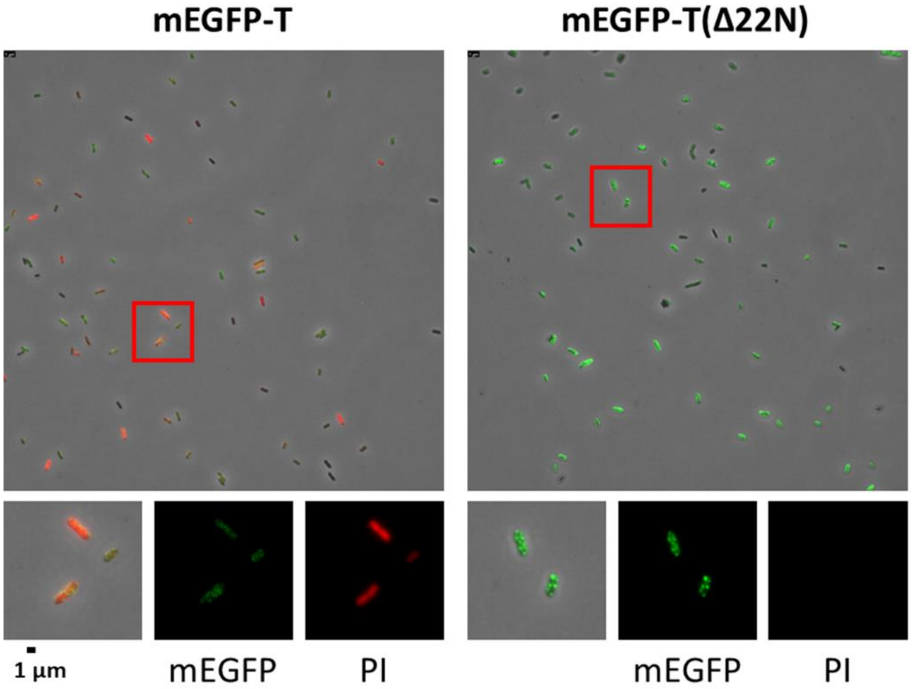

Supplementary Figure 4. The Δ22N truncation abolishes hole formation by holin T. Strains producing for 1 hour mEGFP-fusions of either holin T (left panels) or its N-terminally truncated variant Δ22N (right panels) were stained by propidium iodide (PI), spotted on agarose microscope slides, and analyzed for mEGFP or PI fluorescence, as indicated. No endolysin was present in these strains. Note that the red PI fluorescence is almost absent in case of the holin with the Δ22N truncation. Note also that strongly fluorescent foci are formed with this variant.

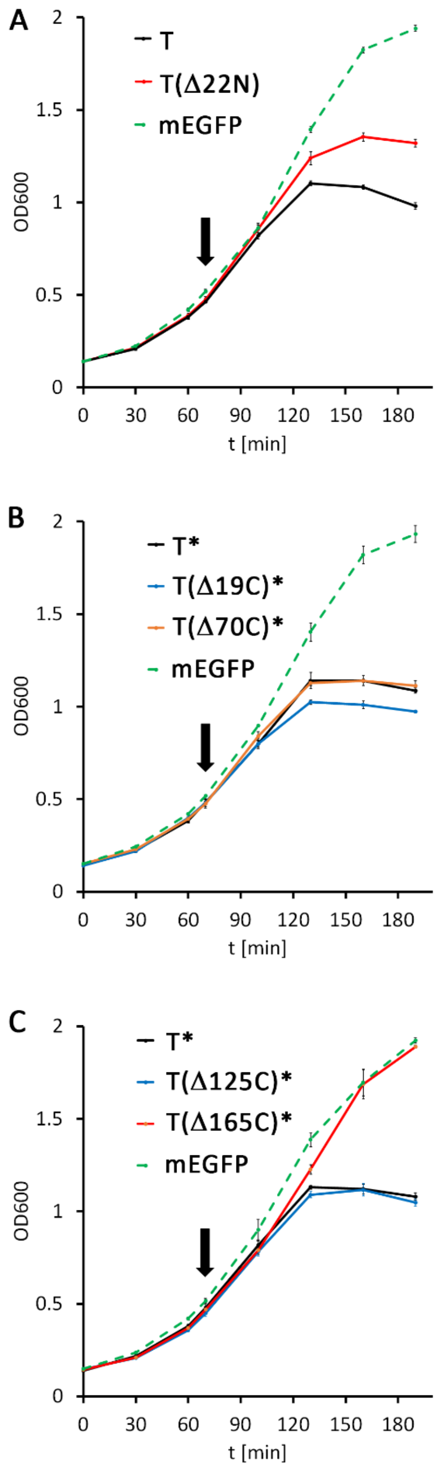

Supplementary Figure 5. In the absence of endolysin, all truncated holin variants with the exception of variant T(Δ165C) cause a growth arrest phenotype. This phenotype likely relates to membrane deenergetization by membrane protein aggregates or pores. mEGFP-fused Holin constructs were the same as in Figures 2 and 3. The asterisks indicate C-terminally FLAG-tagged constructs. For each experiment, the full-length positive control (T or T*) as well as the mEGFP control (without holin) were included.

Full-length holin	...ARILGRAKDYKDDDDK
Δ19C variant	...WYRNDHISDYKDDDDK
Δ70C variant	...EYTVHLNGDYKDDDDK
Δ125C variant	...LQIVHISSDYKDDDDK
Δ165C variant	...IVWYRGDSDYKDDDDK

Supplementary Figure 6. The C-terminal sequences of the FLAG-tagged constructs used for the analysis of C-terminal truncations.

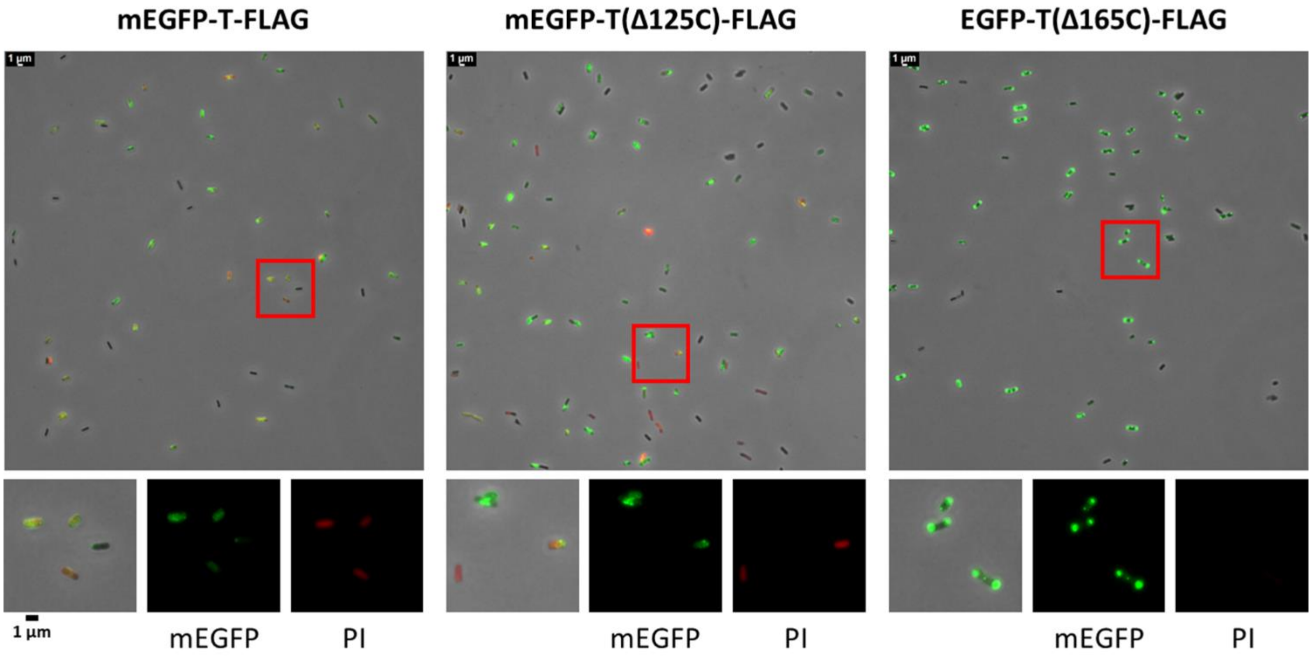

Supplementary Figure 7. The Δ165C truncation abolishes hole formation by holin T. Strains producing for 1 hour mEGFP-fusions of either FLAG-tagged holin T (left panels) or its C-terminally truncated variants Δ125C (middle panels) or Δ165C (right panels) were stained by propidium iodide (PI), spotted on agarose microscope slides, and analyzed for mEGFP or PI fluorescence, as indicated. No endolysin was present in these strains. Note that only in case of the Δ165C construct hole formation was abolished.

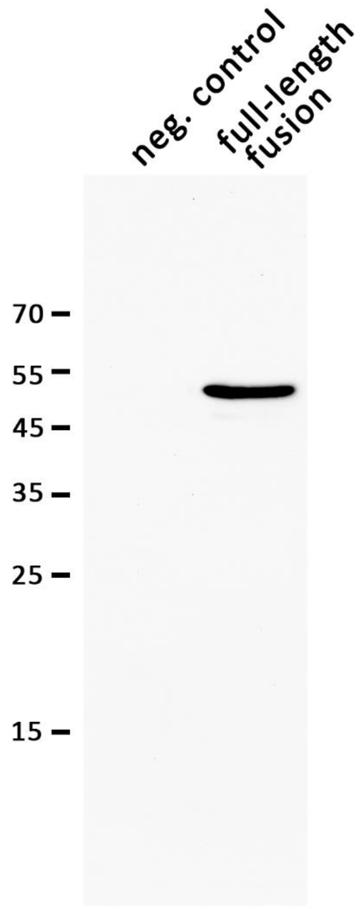

Supplementary Figure 8. There is no N-terminal degradation of the full-length mEGFP-holin fusion, and there is no internal translational start. Western blot of the membrane fraction of strain ER2566 carrying the plasmid pBW-*mEGFP* (negative control) of pBW-*mEGFP-t*-FLAG (full-length fusion), as used for the analysis of C-terminal truncations.

>T
GA**AGGAG**ATATACAT**ATG**GTGAGCAAAGGCGAGGAGCTGTTCACCGGGGTGGTGCCCATCCTGGTCGAGCTGGACGGCGACGTAAACGGCCACAAGTTCAG
CGTGTCCGGCGAGGGCGAGGGCGATGCCACCTACGGCAAGCTGACCCTGAAGTTCATCTGCACCACCGGCAAGCTGCCCGTGCCCTGGCCCACCCTCGTGA
CCACCCTGACCTACGGCGTGCAGTGCTTCAGCCGCTACCCCGACCACATGAAGCAGCACGACTTCTTCAAGTCCGCCATGCCCGAAGGCTACGTCCAGGAG
CGCACCATCTTCTTCAAGGACGACGGCAACTACAAGACCCGCGCCGAGGTGAAGTTCGAGGGCGACACCCTGGTGAACCGCATCGAGCTGAAGGGCATCGA
CTTCAAGGAGGACGGCAACATCCTGGGGCACAAGCTGGAGTACAACTACAACAGCCACAACGTCTATATCATGGCCGACAAGCAGAAGAACGGCATCAAGG
TGAACTTCAAGATCCGCCACAACATCGAGGACGGCAGCGTGCAGCTCGCCGACCACTACCAGCAGAACACCCCCATCGGCGACGGCCCCGTGCTGCTGCCC
GACAACCACTACCTGAGCACCCAGTCCAAGCTGAGCAAAGACCCCAACGAGAAGCGCGATCACATGGTCCTGCTGGAGTTCGTGACCGCCGCCGGGATCAC
TCTCGGCATGGACGAGCTGTACAAGAGACCGGCAGCACCTAGAATATCATTTTCGCCCTCTGATATTCTATTTGGTGTTCTAGATCGCTTGTTCAAAGATA
ACGCTACCGGGAAGGTTCTTGCTTCCCGGGTAGCTGTCGTAATTCTTTTGTTTATAATGGCGATTGTTTGGTATAGGGGAGATAGTTTCTTTGAGTACTAT
AAGCAATCAAAGTATGAAACATACAGTGAAATTATTGAAAAGGAAAGAACTGCACGCTTTGAATCTGTCGCCCTGGAACAACTCCAGATAGTTCATATATC
ATCTGAGGCAGACTTTAGTGCGGTGTATTCTTTCCGCCCTAAAAACTTAAACTATTTTGTTGATATTATAGCATACGAAGGAAAATTACCTTCAACAATAA
GTGAAAAATCACTTGGAGGATATCCTGTTGATAAAACTATGGATGAATATACAGTTCATTTAAATGGACGTCATTATTATTCCAACTCAAAATTTGCTTTT
TTACCAACTAAAAAGCCTACTCCCGAAATAAACTACATGTACAGTTGTCCATATTTTAATTTGGATAATATCTATGCTGGAACGATAACCATGTACTGGTA
TAGAAATGATCATATAAGTAATGACCGCCTTGAATCAATATGTGCTCAGGCGGCCAGAATATTAGGAAGGGCTAAATAA

>T(Δ22N)
GA**AGGAG**ATATACAT**ATG**GTGAGCAAAGGCGAGGAGCTGTTCACCGGGGTGGTGCCCATCCTGGTCGAGCTGGACGGCGACGTAAACGGCCACAAGTTCAG
CGTGTCCGGCGAGGGCGAGGGCGATGCCACCTACGGCAAGCTGACCCTGAAGTTCATCTGCACCACCGGCAAGCTGCCCGTGCCCTGGCCCACCCTCGTGA
CCACCCTGACCTACGGCGTGCAGTGCTTCAGCCGCTACCCCGACCACATGAAGCAGCACGACTTCTTCAAGTCCGCCATGCCCGAAGGCTACGTCCAGGAG
CGCACCATCTTCTTCAAGGACGACGGCAACTACAAGACCCGCGCCGAGGTGAAGTTCGAGGGCGACACCCTGGTGAACCGCATCGAGCTGAAGGGCATCGA
CTTCAAGGAGGACGGCAACATCCTGGGGCACAAGCTGGAGTACAACTACAACAGCCACAACGTCTATATCATGGCCGACAAGCAGAAGAACGGCATCAAGG
TGAACTTCAAGATCCGCCACAACATCGAGGACGGCAGCGTGCAGCTCGCCGACCACTACCAGCAGAACACCCCCATCGGCGACGGCCCCGTGCTGCTGCCC
GACAACCACTACCTGAGCACCCAGTCCAAGCTGAGCAAAGACCCCAACGAGAAGCGCGATCACATGGTCCTGCTGGAGTTCGTGACCGCCGCCGGGATCAC
TCTCGGCATGGACGAGCTGTACAAGAAAGATAACGCTACCGGGAAGGTTCTTGCTTCCCGGGTAGCTGTCGTAATTCTTTTGTTTATAATGGCGATTGTTT
GGTATAGGGGAGATAGTTTCTTTGAGTACTATAAGCAATCAAAGTATGAAACATACAGTGAAATTATTGAAAAGGAAAGAACTGCACGCTTTGAATCTGTC
GCCCTGGAACAACTCCAGATAGTTCATATATCATCTGAGGCAGACTTTAGTGCGGTGTATTCTTTCCGCCCTAAAAACTTAAACTATTTTGTTGATATTAT
AGCATACGAAGGAAAATTACCTTCAACAATAAGTGAAAAATCACTTGGAGGATATCCTGTTGATAAAACTATGGATGAATATACAGTTCATTTAAATGGAC
GTCATTATTATTCCAACTCAAAATTTGCTTTTTTACCAACTAAAAAGCCTACTCCCGAAATAAACTACATGTACAGTTGTCCATATTTTAATTTGGATAAT
ATCTATGCTGGAACGATAACCATGTACTGGTATAGAAATGATCATATAAGTAATGACCGCCTTGAATCAATATGTGCTCAGGCGGCCAGAATATTAGGAAG
GGCTAAATAA

>T(F22A)
GA**AGGAG**ATATACAT**ATG**GTGAGCAAAGGCGAGGAGCTGTTCACCGGGGTGGTGCCCATCCTGGTCGAGCTGGACGGCGACGTAAACGGCCACAAGTTCAG
CGTGTCCGGCGAGGGCGAGGGCGATGCCACCTACGGCAAGCTGACCCTGAAGTTCATCTGCACCACCGGCAAGCTGCCCGTGCCCTGGCCCACCCTCGTGA
CCACCCTGACCTACGGCGTGCAGTGCTTCAGCCGCTACCCCGACCACATGAAGCAGCACGACTTCTTCAAGTCCGCCATGCCCGAAGGCTACGTCCAGGAG
CGCACCATCTTCTTCAAGGACGACGGCAACTACAAGACCCGCGCCGAGGTGAAGTTCGAGGGCGACACCCTGGTGAACCGCATCGAGCTGAAGGGCATCGA
CTTCAAGGAGGACGGCAACATCCTGGGGCACAAGCTGGAGTACAACTACAACAGCCACAACGTCTATATCATGGCCGACAAGCAGAAGAACGGCATCAAGG
TGAACTTCAAGATCCGCCACAACATCGAGGACGGCAGCGTGCAGCTCGCCGACCACTACCAGCAGAACACCCCCATCGGCGACGGCCCCGTGCTGCTGCCC
GACAACCACTACCTGAGCACCCAGTCCAAGCTGAGCAAAGACCCCAACGAGAAGCGCGATCACATGGTCCTGCTGGAGTTCGTGACCGCCGCCGGGATCAC
TCTCGGCATGGACGAGCTGTACAAGAGACCGGCAGCACCTAGAATATCATTTTCGCCCTCTGATATTCTATTTGGTGTTCTAGATCGCTTGGCCAAAGATA
ACGCTACCGGGAAGGTTCTTGCTTCCCGGGTAGCTGTCGTAATTCTTTTGTTTATAATGGCGATTGTTTGGTATAGGGGAGATAGTTTCTTTGAGTACTAT
AAGCAATCAAAGTATGAAACATACAGTGAAATTATTGAAAAGGAAAGAACTGCACGCTTTGAATCTGTCGCCCTGGAACAACTCCAGATAGTTCATATATC
ATCTGAGGCAGACTTTAGTGCGGTGTATTCTTTCCGCCCTAAAAACTTAAACTATTTTGTTGATATTATAGCATACGAAGGAAAATTACCTTCAACAATAA
GTGAAAAATCACTTGGAGGATATCCTGTTGATAAAACTATGGATGAATATACAGTTCATTTAAATGGACGTCATTATTATTCCAACTCAAAATTTGCTTTT
TTACCAACTAAAAAGCCTACTCCCGAAATAAACTACATGTACAGTTGTCCATATTTTAATTTGGATAATATCTATGCTGGAACGATAACCATGTACTGGTA
TAGAAATGATCATATAAGTAATGACCGCCTTGAATCAATATGTGCTCAGGCGGCCAGAATATTAGGAAGGGCTAAATAA

>T(R34A)
GA**AGGAG**ATATACAT**ATG**GTGAGCAAAGGCGAGGAGCTGTTCACCGGGGTGGTGCCCATCCTGGTCGAGCTGGACGGCGACGTAAACGGCCACAAGTTCAG
CGTGTCCGGCGAGGGCGAGGGCGATGCCACCTACGGCAAGCTGACCCTGAAGTTCATCTGCACCACCGGCAAGCTGCCCGTGCCCTGGCCCACCCTCGTGA
CCACCCTGACCTACGGCGTGCAGTGCTTCAGCCGCTACCCCGACCACATGAAGCAGCACGACTTCTTCAAGTCCGCCATGCCCGAAGGCTACGTCCAGGAG
CGCACCATCTTCTTCAAGGACGACGGCAACTACAAGACCCGCGCCGAGGTGAAGTTCGAGGGCGACACCCTGGTGAACCGCATCGAGCTGAAGGGCATCGA
CTTCAAGGAGGACGGCAACATCCTGGGGCACAAGCTGGAGTACAACTACAACAGCCACAACGTCTATATCATGGCCGACAAGCAGAAGAACGGCATCAAGG
TGAACTTCAAGATCCGCCACAACATCGAGGACGGCAGCGTGCAGCTCGCCGACCACTACCAGCAGAACACCCCCATCGGCGACGGCCCCGTGCTGCTGCCC
GACAACCACTACCTGAGCACCCAGTCCAAGCTGAGCAAAGACCCCAACGAGAAGCGCGATCACATGGTCCTGCTGGAGTTCGTGACCGCCGCCGGGATCAC
TCTCGGCATGGACGAGCTGTACAAGAGACCGGCAGCACCTAGAATATCATTTTCGCCCTCTGATATTCTATTTGGTGTTCTAGATCGCTTGTTCAAAGATA
ACGCTACCGGGAAGGTTCTTGCTTCCGCGGTAGCTGTCGTAATTCTTTTGTTTATAATGGCGATTGTTTGGTATAGGGGAGATAGTTTCTTTGAGTACTAT
AAGCAATCAAAGTATGAAACATACAGTGAAATTATTGAAAAGGAAAGAACTGCACGCTTTGAATCTGTCGCCCTGGAACAACTCCAGATAGTTCATATATC
ATCTGAGGCAGACTTTAGTGCGGTGTATTCTTTCCGCCCTAAAAACTTAAACTATTTTGTTGATATTATAGCATACGAAGGAAAATTACCTTCAACAATAA
GTGAAAAATCACTTGGAGGATATCCTGTTGATAAAACTATGGATGAATATACAGTTCATTTAAATGGACGTCATTATTATTCCAACTCAAAATTTGCTTTT
TTACCAACTAAAAAGCCTACTCCCGAAATAAACTACATGTACAGTTGTCCATATTTTAATTTGGATAATATCTATGCTGGAACGATAACCATGTACTGGTA
TAGAAATGATCATATAAGTAATGACCGCCTTGAATCAATATGTGCTCAGGCGGCCAGAATATTAGGAAGGGCTAAATAA

> T* (*full-length holin with FLAG-tag, used as control for analyses of C-terminal truncations)
GA**AGGAG**ATATACAT**ATG**GTGAGCAAAGGCGAGGAGCTGTTCACCGGGGTGGTGCCCATCCTGGTCGAGCTGGACGGCGACGTAAACGGCCACAAGTTCAG
CGTGTCCGGCGAGGGCGAGGGCGATGCCACCTACGGCAAGCTGACCCTGAAGTTCATCTGCACCACCGGCAAGCTGCCCGTGCCCTGGCCCACCCTCGTGA
CCACCCTGACCTACGGCGTGCAGTGCTTCAGCCGCTACCCCGACCACATGAAGCAGCACGACTTCTTCAAGTCCGCCATGCCCGAAGGCTACGTCCAGGAG
CGCACCATCTTCTTCAAGGACGACGGCAACTACAAGACCCGCGCCGAGGTGAAGTTCGAGGGCGACACCCTGGTGAACCGCATCGAGCTGAAGGGCATCGA
CTTCAAGGAGGACGGCAACATCCTGGGGCACAAGCTGGAGTACAACTACAACAGCCACAACGTCTATATCATGGCCGACAAGCAGAAGAACGGCATCAAGG
TGAACTTCAAGATCCGCCACAACATCGAGGACGGCAGCGTGCAGCTCGCCGACCACTACCAGCAGAACACCCCCATCGGCGACGGCCCCGTGCTGCTGCCC
GACAACCACTACCTGAGCACCCAGTCCAAGCTGAGCAAAGACCCCAACGAGAAGCGCGATCACATGGTCCTGCTGGAGTTCGTGACCGCCGCCGGGATCAC
TCTCGGCATGGACGAGCTGTACAAGAGACCGGCAGCACCTAGAATATCATTTTCGCCCTCTGATATTCTATTTGGTGTTCTAGATCGCTTGTTCAAAGATA
ACGCTACCGGGAAGGTTCTTGCTTCCCGGGTAGCTGTCGTAATTCTTTTGTTTATAATGGCGATTGTTTGGTATAGGGGAGATAGTTTCTTTGAGTACTAT
AAGCAATCAAAGTATGAAACATACAGTGAAATTATTGAAAAGGAAAGAACTGCACGCTTTGAATCTGTCGCCCTGGAACAACTCCAGATAGTTCATATATC
ATCTGAGGCAGACTTTAGTGCGGTGTATTCTTTCCGCCCTAAAAACTTAAACTATTTTGTTGATATTATAGCATACGAAGGAAAATTACCTTCAACAATAA

GTGAAAATCACTTGGAGGATATCCTGTTGATAAAACTATGGATGAATATACAGTTCATTTAAATGGACGTCATTATTATTCCAACTCAAAATTTGCTTTT
TTACCAACTAAAAAGCCTACTCCCGAAATAAACTACATGTACAGTTGTCCATATTTTAATTTGGATAATATCTATGCTGGAACGATAACCATGTACTGGTA
TAGAAATGATCATATAAGTAATGACCGCCTTGAATCAATATGTGCTCAGGCGGCCAGAATATTAGGAAGGGCTAAAGACTACAAAGACGATGACGACAAGT
AA

>T(Δ19C)

GA**AGGAG**ATATACAT**ATG**GTGAGCAAAGGCGAGGAGCTGTTCACCGGGGTGGTGCCCATCCTGGTCGAGCTGGACGGCGACGTAAACGGCCACAAGTTCAG
CGTGTCCGGCGAGGGCGAGGGCGATGCCACCTACGGCAAGCTGACCCTGAAGTTCATCTGCACCACCGGCAAGCTGCCCGTGCCCTGGCCCACCCTCGTGA
CCACCCTGACCTACGGCGTGCAGTGCTTCAGCCGCTACCCCGACCACATGAAGCAGCACGACTTCTTCAAGTCCGCCATGCCCGAAGGCTACGTCCAGGAG
CGCACCATCTTCTTCAAGGACGACGGCAACTACAAGACCCGCGCCGAGGTGAAGTTCGAGGGCGACACCCTGGTGAACCGCATCGAGCTGAAGGGCATCGA
CTTCAAGGAGGACGGCAACATCCTGGGGCACAAGCTGGAGTACAACTACAACAGCCACAACGTCTATATCATGGCCGACAAGCAGAAGAACGGCATCAAGG
TGAACTTCAAGATCCGCCACAACATCGAGGACGGCAGCGTGCAGCTCGCCGACCACTACCAGCAGAACACCCCCATCGGCGACGGCCCCGTGCTGCTGCCC
GACAACCACTACCTGAGCACCCAGTCCAAGCTGAGCAAAGACCCCAACGAGAAGCGCGATCACATGGTCCTGCTGGAGTTCGTGACCGCCGCCGGGATCAC
TCTCGGCATGGACGAGCTGTACAAGAGACCGGCAGCACCTAGAATATCATTTTCGCCCTCTGATATTCTATTTGGTGTTCTAGATCGCTTGTTCAAAGATA
ACGCTACCGGGAAGGTTCTTGCTTCCCGGGTAGCTGTCGTAATTCTTTTGTTTATAATGGCGATTGTTTGGTATAGGGGAGATAGTTTCTTTGAGTACTAT
AAGCAATCAAAGTATGAAACATACAGTGAAATTATTGAAAAGGAAAGAACTGCACGCTTTGAATCTGTCGCCCTGGAACAACTCCAGATAGTTCATATATC
ATCTGAGGCAGACTTTAGTGCGGTGTATTCTTTCCGCCCTAAAAACTTAAACTATTTTGTTGATATTATAGCATACGAAGGAAAATTACCTTCAACAATAA
GTGAAAATCACTTGGAGGATATCCTGTTGATAAAACTATGGATGAATATACAGTTCATTTAAATGGACGTCATTATTATTCCAACTCAAAATTTGCTTTT
TTACCAACTAAAAAGCCTACTCCCGAAATAAACTACATGTACAGTTGTCCATATTTTAATTTGGATAATATCTATGCTGGAACGATAACCATGTACTGGTA
TAGAAATGATCATATAAGTGACTACAAAGACGATGACGACAAGTAA

>T(Δ70C)

GA**AGGAG**ATATACAT**ATG**GTGAGCAAAGGCGAGGAGCTGTTCACCGGGGTGGTGCCCATCCTGGTCGAGCTGGACGGCGACGTAAACGGCCACAAGTTCAG
CGTGTCCGGCGAGGGCGAGGGCGATGCCACCTACGGCAAGCTGACCCTGAAGTTCATCTGCACCACCGGCAAGCTGCCCGTGCCCTGGCCCACCCTCGTGA
CCACCCTGACCTACGGCGTGCAGTGCTTCAGCCGCTACCCCGACCACATGAAGCAGCACGACTTCTTCAAGTCCGCCATGCCCGAAGGCTACGTCCAGGAG
CGCACCATCTTCTTCAAGGACGACGGCAACTACAAGACCCGCGCCGAGGTGAAGTTCGAGGGCGACACCCTGGTGAACCGCATCGAGCTGAAGGGCATCGA
CTTCAAGGAGGACGGCAACATCCTGGGGCACAAGCTGGAGTACAACTACAACAGCCACAACGTCTATATCATGGCCGACAAGCAGAAGAACGGCATCAAGG
TGAACTTCAAGATCCGCCACAACATCGAGGACGGCAGCGTGCAGCTCGCCGACCACTACCAGCAGAACACCCCCATCGGCGACGGCCCCGTGCTGCTGCCC
GACAACCACTACCTGAGCACCCAGTCCAAGCTGAGCAAAGACCCCAACGAGAAGCGCGATCACATGGTCCTGCTGGAGTTCGTGACCGCCGCCGGGATCAC
TCTCGGCATGGACGAGCTGTACAAGAGACCGGCAGCACCTAGAATATCATTTTCGCCCTCTGATATTCTATTTGGTGTTCTAGATCGCTTGTTCAAAGATA
ACGCTACCGGGAAGGTTCTTGCTTCCCGGGTAGCTGTCGTAATTCTTTTGTTTATAATGGCGATTGTTTGGTATAGGGGAGATAGTTTCTTTGAGTACTAT
AAGCAATCAAAGTATGAAACATACAGTGAAATTATTGAAAAGGAAAGAACTGCACGCTTTGAATCTGTCGCCCTGGAACAACTCCAGATAGTTCATATATC
ATCTGAGGCAGACTTTAGTGCGGTGTATTCTTTCCGCCCTAAAAACTTAAACTATTTTGTTGATATTATAGCATACGAAGGAAAATTACCTTCAACAATAA
GTGAAAATCACTTGGAGGATATCCTGTTGATAAAACTATGGATGAATATACAGTTCATTTAAATGGAGACTACAAAGACGATGACGACAAGTAA

>T(Δ125C)

GA**AGGAG**ATATACAT**ATG**GTGAGCAAAGGCGAGGAGCTGTTCACCGGGGTGGTGCCCATCCTGGTCGAGCTGGACGGCGACGTAAACGGCCACAAGTTCAG
CGTGTCCGGCGAGGGCGAGGGCGATGCCACCTACGGCAAGCTGACCCTGAAGTTCATCTGCACCACCGGCAAGCTGCCCGTGCCCTGGCCCACCCTCGTGA
CCACCCTGACCTACGGCGTGCAGTGCTTCAGCCGCTACCCCGACCACATGAAGCAGCACGACTTCTTCAAGTCCGCCATGCCCGAAGGCTACGTCCAGGAG
CGCACCATCTTCTTCAAGGACGACGGCAACTACAAGACCCGCGCCGAGGTGAAGTTCGAGGGCGACACCCTGGTGAACCGCATCGAGCTGAAGGGCATCGA
CTTCAAGGAGGACGGCAACATCCTGGGGCACAAGCTGGAGTACAACTACAACAGCCACAACGTCTATATCATGGCCGACAAGCAGAAGAACGGCATCAAGG
TGAACTTCAAGATCCGCCACAACATCGAGGACGGCAGCGTGCAGCTCGCCGACCACTACCAGCAGAACACCCCCATCGGCGACGGCCCCGTGCTGCTGCCC
GACAACCACTACCTGAGCACCCAGTCCAAGCTGAGCAAAGACCCCAACGAGAAGCGCGATCACATGGTCCTGCTGGAGTTCGTGACCGCCGCCGGGATCAC
TCTCGGCATGGACGAGCTGTACAAGAGACCGGCAGCACCTAGAATATCATTTTCGCCCTCTGATATTCTATTTGGTGTTCTAGATCGCTTGTTCAAAGATA
ACGCTACCGGGAAGGTTCTTGCTTCCCGGGTAGCTGTCGTAATTCTTTTGTTTATAATGGCGATTGTTTGGTATAGGGGAGATAGTTTCTTTGAGTACTAT
AAGCAATCAAAGTATGAAACATACAGTGAAATTATTGAAAAGGAAAGAACTGCACGCTTTGAATCTGTCGCCCTGGAACAACTCCAGATAGTTCATATATC
ATCTGACTACAAAGACGATGACGACAAGTAA

>T(Δ165C)

GA**AGGAG**ATATACAT**ATG**GTGAGCAAAGGCGAGGAGCTGTTCACCGGGGTGGTGCCCATCCTGGTCGAGCTGGACGGCGACGTAAACGGCCACAAGTTCAG
CGTGTCCGGCGAGGGCGAGGGCGATGCCACCTACGGCAAGCTGACCCTGAAGTTCATCTGCACCACCGGCAAGCTGCCCGTGCCCTGGCCCACCCTCGTGA
CCACCCTGACCTACGGCGTGCAGTGCTTCAGCCGCTACCCCGACCACATGAAGCAGCACGACTTCTTCAAGTCCGCCATGCCCGAAGGCTACGTCCAGGAG
CGCACCATCTTCTTCAAGGACGACGGCAACTACAAGACCCGCGCCGAGGTGAAGTTCGAGGGCGACACCCTGGTGAACCGCATCGAGCTGAAGGGCATCGA
CTTCAAGGAGGACGGCAACATCCTGGGGCACAAGCTGGAGTACAACTACAACAGCCACAACGTCTATATCATGGCCGACAAGCAGAAGAACGGCATCAAGG
TGAACTTCAAGATCCGCCACAACATCGAGGACGGCAGCGTGCAGCTCGCCGACCACTACCAGCAGAACACCCCCATCGGCGACGGCCCCGTGCTGCTGCCC
GACAACCACTACCTGAGCACCCAGTCCAAGCTGAGCAAAGACCCCAACGAGAAGCGCGATCACATGGTCCTGCTGGAGTTCGTGACCGCCGCCGGGATCAC
TCTCGGCATGGACGAGCTGTACAAGAGACCGGCAGCACCTAGAATATCATTTTCGCCCTCTGATATTCTATTTGGTGTTCTAGATCGCTTGTTCAAAGATA
ACGCTACCGGGAAGGTTCTTGCTTCCCGGGTAGCTGTCGTAATTCTTTTGTTTATAATGGCGATTGTTTGGTATAGGGGAGATAGTGACTACAAAGACGAT
GACGACAAGTAA

>mEGFP (negative control without holin)

GA**AGGAG**ATATACAT**ATG**GTGAGCAAAGGCGAGGAGCTGTTCACCGGGGTGGTGCCCATCCTGGTCGAGCTGGACGGCGACGTAAACGGCCACAAGTTCAG
CGTGTCCGGCGAGGGCGAGGGCGATGCCACCTACGGCAAGCTGACCCTGAAGTTCATCTGCACCACCGGCAAGCTGCCCGTGCCCTGGCCCACCCTCGTGA
CCACCCTGACCTACGGCGTGCAGTGCTTCAGCCGCTACCCCGACCACATGAAGCAGCACGACTTCTTCAAGTCCGCCATGCCCGAAGGCTACGTCCAGGAG
CGCACCATCTTCTTCAAGGACGACGGCAACTACAAGACCCGCGCCGAGGTGAAGTTCGAGGGCGACACCCTGGTGAACCGCATCGAGCTGAAGGGCATCGA
CTTCAAGGAGGACGGCAACATCCTGGGGCACAAGCTGGAGTACAACTACAACAGCCACAACGTCTATATCATGGCCGACAAGCAGAAGAACGGCATCAAGG
TGAACTTCAAGATCCGCCACAACATCGAGGACGGCAGCGTGCAGCTCGCCGACCACTACCAGCAGAACACCCCCATCGGCGACGGCCCCGTGCTGCTGCCC
GACAACCACTACCTGAGCACCCAGTCCAAGCTGAGCAAAGACCCCAACGAGAAGCGCGATCACATGGTCCTGCTGGAGTTCGTGACCGCCGCCGGGATCAC
TCTCGGCATGGACGAGCTGTACAAGTAA

Supplementary Figure 9. DNA sequences of mEGFP-T fusions and its variants used in this study. The ribosomal binding site and translational start codon are highlighted (bold, underlined). Green, mEGFP-coding regions; red, holin-coding regions; blue: FLAG-tag-coding regions; Note that no internal methionine is left at the fusion position, and that mEGFP is connected to the holin via natively unfolded regions (underlined).

```
T4          ----MAAPRI-SFSPSDILFGVLDRLFKDNATGKVLASRVAVVILLFIMAIVWYRGDSFF     55
JS98        -----MEPKV-GISIPDLLFGLLDRIFKDNATGKVVFSRVLVIVLLFLMAVIWYKGDALL     54
RB69        MGGNMAAPKV-SFSPSDILFGLLDRIFKDNASGNILISRVAVVVLLFLMALIWYKGNIFM     59
RB49        -------MQVSEKGKDFAISNVLRAIFTTKS-TELLVLRVFAAVVLSILAFVVYSKNELF     52
44RR2       MSNQPTKTENQTGGRAGVLMDILDRLFKDAVTGEIVFYRAILLTLVFLMGFSWYSKEEIF     60
                    .    .    : .:*  :*.     :::  *.    :: ::..  *  : ::

T4          EYYKQSKYETYSEIIEKERTARFESVALEQLQIVHISSEADFSAVYSFRPKNLNYFVDII     115
JS98        DAYRDASYASYTEMIRQDQDNRFKIAAIEQIQIVHSSSGADFTAIYSFRPTNLNYFVDMV     114
RB69        DYYVRSKYDTYTEVIQKERNTRFESAALEQLQIVHVTSRADFSSVYSFRPKNLNYFVDLI     119
RB49        ALYKETRYETYAHILQVEKDRNFDNAAQEQLQIVHVSADADFSAVFSFRPKNLNYFVDLV     112
44RR2       ALYKETRFENYQEILQAERDRKFEMAAQEQLQIAHVSSRADFSVVFSFRPRNMNYFVDMM     120
              *  : : .* .::. ::  .*. .* **:**.* :: ***: ::**** *:*****::

T4          AYEGKLPSTISEKSLGGYPVDKTMDEYTVHLNGRHYYSNSKFAFLPTKKPT---PEINYM     172
JS98        GYEGILPDTVDEKNLGGFPIDKTSAEYLAGVNGNYFESSTESVFLPTKKK----SSFAYM     170
RB69        AYEGKLPSTVTEKSMGGFPVDKTTAEYSVHLSGLHFTSKTDFAFLPTKSKT---PELAYM     176
RB49        AYEGKLPHTIDEKNLGGFPINKTSEEYRRHLLGKSYFTDKDFQYIPSREKKLENIDIGFM     172
44RR2       ATEGKTPTDLIGREKGGYPINKTSNEYMVHMSGRHFSNYKEFAYLPAGHE-----DFEYM     175
            . **  *  :  :. **:*::**  **   : *  : . ..  ::*:        .: :*

T4          YSCPYFNLDNIYAGTITMYWYRNDH-ISNDRLESICAQAARILGRAK  218
JS98        FSCPFFNLENVYAGTVSLYWYKQKPNLGFDRLSAMCGQAGRTLGRTR  217
RB69        YSCPYFNLDNIYAGTVSMYWYKGSDVLNEERLAAICNQAARILGRAK  223
RB49        YSCPIFNLDNVYSGSIAISWK-NKPDIDIENLDTLCNQSARILGRIR  218
44RR2       YSCPITNLDNIYAGSVSMFWK-KKPVINENKLFVICNQAERLLSRAR  221
            :***  **:*:*:*:::: *   .  :. :.*  :* *: * *.* :
```

Supplementary Figure 10. Alignment of closely related holin homologs from phages T4, JS98, RB69, RB49, and 44RR2. The N-terminal amphipathic helix is highlighted in blue, the transmembrane helix in yellow, the bridge helix in green, and the C-terminal globular domain is underlined. Note that most conserved regions are in the C-terminal globular domain, which mediates interactions with antiholin RI and stabilizes holin-holin self interactions. In the N-terminal domain, which is essential for hole formation, only few residues are highly conserved, including F22 and R34.

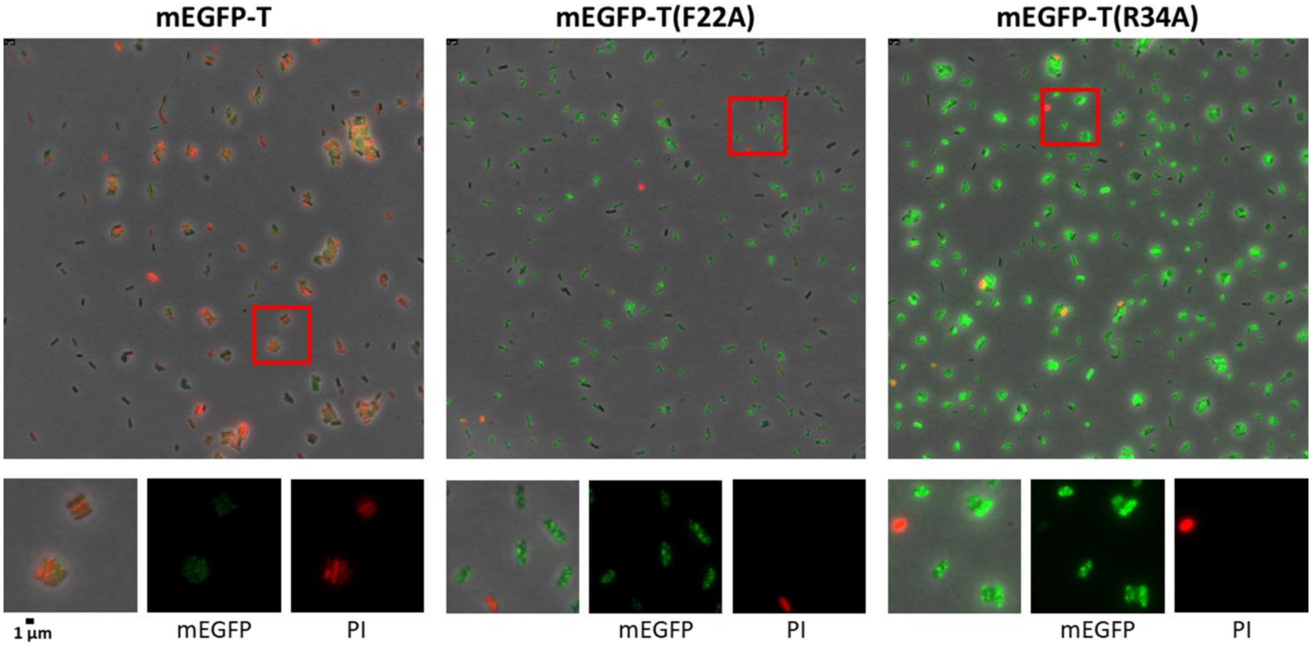

Supplementary Figure 11. The F22A and R34A substitutions in holin T do not form functional holes. Strains producing for 1 hour mEGFP-fusions of either holin T (left panels) or its F22A or R34A variants (middle and right panels, respecively) were stained by propidium iodide (PI), spotted on agarose microscope slides, and analyzed for mEGFP or PI fluorescence, as indicated. No endolysin was present in these strains. Note that the F22A and R34A substitutions both markedly reduce the abundance of PI-stainable cells, and more mEGFP-fused holin can be made with the mutated variants, likely due to intact membranes (no holes) and consequently better cellular energetization. Even the high load with the mutated mEGFP-tagged holin variants did not result in membrane permeabilization, and the few stained cells in these experiment usually were dead without significant mEGFP signal.

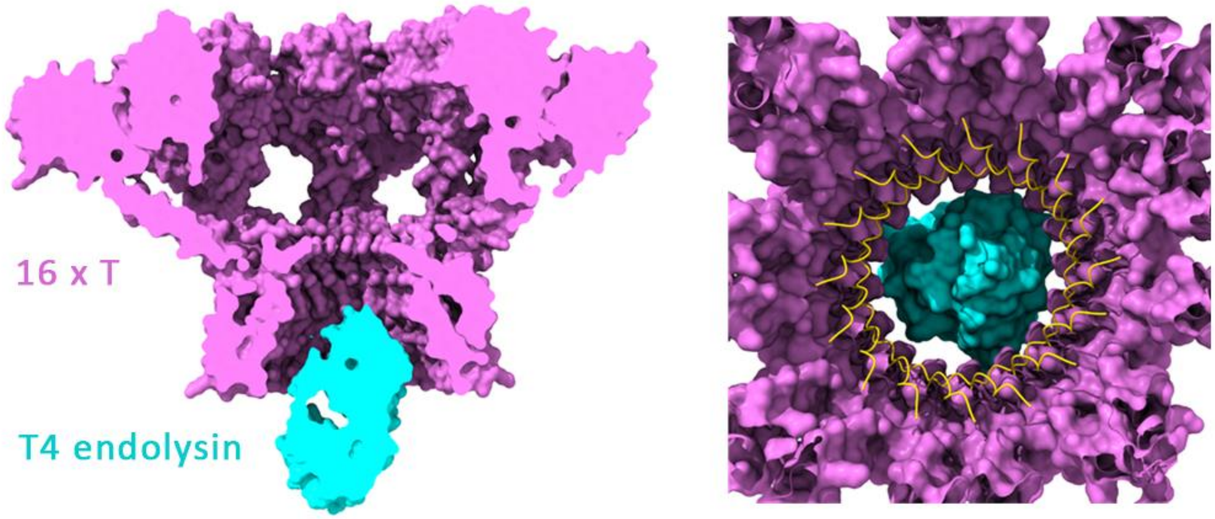

Supplementary Figure 12. The T4 endolysin would fit into the hydrophilic hole generated by 16 T4 holins. The holin ring was modeled by AlphaFold 3. The endolysin structure (T4 lysozyme) was taken from PDB 148L (Kuroki et al. 1993). Note that an apparent single constriction in the pore is due to an arginine in the highly flexible N-terminal region. The right image shows the flexible unstructured N-terminal region as yellow backbone, indicating that the pore would suffice for the endolysin passage.
